# The effect of high perioperative inspiratory oxygen fraction for abdominal surgery on surgical site infection: a systematic review and meta-analysis

**DOI:** 10.1038/s41598-023-41300-4

**Published:** 2023-09-20

**Authors:** Jae Hee Kuh, Woo-Seok Jung, Leerang Lim, Hae Kyung Yoo, Jae-Woo Ju, Ho-Jin Lee, Won Ho Kim

**Affiliations:** Department of Anesthesiology and Pain Medicine, Seoul National University Hospital, Seoul National University College of Medicine, 101 Daehak-Ro, Jongno-Gu, Seoul, 03080 Republic of Korea

**Keywords:** Gastrointestinal diseases, Risk factors

## Abstract

Guidelines from the World Health Organization strongly recommend the use of a high fraction of inspired oxygen (FiO_2_) in adult patients undergoing general anesthesia to reduce surgical site infection (SSI). However, previous meta-analyses reported inconsistent results. We aimed to address this controversy by focusing specifically on abdominal surgery with relatively high risk of SSI. Medline, EMBASE, and Cochrane CENTRAL databases were searched. Randomized trials of abdominal surgery comparing high to low perioperative FiO_2_ were included, given that the incidence of SSI was reported as an outcome. Meta-analyses of risk ratios (RR) were performed using a fixed effects model. Subgroup analysis and meta-regression were employed to explore sources of heterogeneity. We included 27 trials involving 15977 patients. The use of high FiO_2_ significantly reduced the incidence of SSI (n = 27, risk ratio (RR): 0.87; 95% confidence interval (CI): 0.79, 0.95; I^2^ = 49%, Z = 3.05). Trial sequential analysis (TSA) revealed that z-curve crossed the trial sequential boundary and data are sufficient. This finding held true for the subgroup of emergency operations (n = 2, RR: 0.54; 95% CI: 0.35, 0.84; I^2^ = 0%, Z = 2.75), procedures using air as carrier gas (n = 9, RR: 0.79; 95% CI: 0.69, 0.91; I^2^ = 60%, Z = 3.26), and when a high level of FiO_2_ was maintained for a postoperative 6 h or more (n = 9, RR: 0.68; 95% CI: 0.56, 0.83; I^2^ = 46%, Z = 3.83). Meta-regression revealed no significant interaction between SSI with any covariates including age, sex, body-mass index, diabetes mellitus, duration of surgery, and smoking. Quality of evidence was assessed to be moderate to very low. Our pooled analysis revealed that the application of high FiO_2_ reduced the incidence of SSI after abdominal operations. Although TSA demonstrated sufficient data and cumulative analysis crossed the TSA boundary, our results should be interpreted cautiously given the low quality of evidence.

**Registration**: https://www.crd.york.ac.uk/prospero (CRD42022369212) on October 2022.

## Introduction

Millions of patients undergo surgery under general anesthesia each year^1^. Anesthesiologists optimize ventilator setting to reduce postoperative morbidity and mortality and the fraction of inspired oxygen (FiO_2_) is one of the key settings. Surgical site infection (SSI) is a common and serious complication after abdominal surgery^2^. World Health Organization (WHO) implemented guidelines recommending the use of high fraction of inspired oxygen (FiO_2_) during the perioperative period to reduce the risk of surgical site infection (SSI)^3^. Poor quality of evidence and failure to address potential harms^4^ led to an updated analysis^5^, limiting the intervention to adult patients undergoing general anesthesia. Currently, the US Centers for Disease Control and Prevention (CDC) and other healthcare organizations have adopted the revised WHO standards^6,7^. The consensus is that administering high FiO_2_ and thereby increasing tissue oxygen tension^8^ could lower SSI by mechanisms such as facilitating neutrophil bacterial killing^9^. Additional benefits such as the reduction of postoperative nausea and vomiting have been debated^10,11^.

Nonetheless, concerns still prevail. The current recommendations yet undermine the known adverse effects of high FiO_2_—among the notable drawbacks lies absorptive atelectasis^12^, which potentially results in decreased lung compliance and impaired oxygenation, as well as pneumonia^13^. It is further hypothesized that FiO_2_ above physiological range imposes systematic oxidative stress; this may lead to respiratory and cardiovascular complications^13,14^, neurological manifestations^15^, and death. Acutely ill patients fare better with conservative oxygen therapy^16,17^, accordingly, high FiO_2_ is not recommended in emergency operations or for critical care patients^18,19^.

There have been attempts to tackle this debate. While older studies focused on the prevention of either SSI or postoperative nausea and vomiting^20^, recent meta-analyses reported diverse clinical effects such as length of hospital stay and mortality^21–23^. Despite the increase in scope, previous reviews have neglected to analyze the substantial differences arising from surgical or patient characteristics. For instance, limiting included trials to those operated under general anesthesia prohibited the exclusion of the effect of general anesthetics on wound infection. General anesthesia corresponded to a higher risk of SSI^24^. Furthermore, open surgical approach and emergency operations^25^, male sex, and length of the procedure^26^ were documented as independent risk factors of SSI.

We believe that surgery type is also a critical factor associated with SSI incidence and that different types should be investigated separately. Previous trials of abdominal surgery indicated a high incidence of 15–25%^27,28^. SSI remains an unrelenting source of morbidity for colorectal operations with an incidence rate of 9.34%^29^, or pancreatoduodenectomy with an incidence of 6–17%^30^. A recent trial of emergency abdominal surgery showed that perioperative administration of 80% FiO_2_ significantly decreased SSI incidence^31^. In addition, previous randomized trials reported the significant positive effect of high FiO_2_ on SSI in abdominal surgeries^32,33^. However, a meta-analysis focusing solely on abdominal surgery has rarely been conducted to the best of our knowledge. ‘

Therefore, we carried out a meta-analysis on the effect of high FiO_2_ on SSI and other clinical outcomes focusing only on abdominal surgeries; we aimed to discover causes of heterogeneity unexposed by previous reviews by performing subgroup analysis or meta-regression for the important risk factors previously reported.

## Methods

Following the registration on PROSPERO (https://www.crd.york.ac.uk/prospero, registration number: CRD42022369212) on October 2022, the present study was conducted according to the recommendations of the Cochrane Handbook for Systematic Reviews of Interventions. The reporting of this review follows the Preferred Reporting Items for Systematic Reviews and Meta-Analyses (PRISMA) statement guidelines.

A search strategy was developed and applied to Medline, Embase, and Cochrane CENTRAL, and is provided in the protocol (Supplemental Text S1). The inclusion and exclusion criteria of our review was reported in Supplemental Text S2. The last search was executed on October 21st, 2022. Only full-text articles in English were considered as candidates.

Two authors (KJH and WHK) independently screened titles and abstracts for relevant trials. The full text of manuscripts that passed the first level of screening was scrutinized to determine eligibility. Studies were qualified for inclusion if they: (1) mentioned surgical site infection (SSI) incidence following abdominal surgery; (2) compared high FiO_2_ of at least 80% with standard levels of at most 40%; and (3) were randomized controlled trials. The references of previous meta-analyses and related articles were manually inspected to incorporate any studies omitted in the original search. Any disagreements were resolved via discussion.

We piloted a standardized extraction form which one author (JHK) filled out and another (WHK) confirmed. Data was reexamined several times to amend any remaining errors. The following data were extracted: study design, inclusion and exclusion criteria, surgery type, the urgency of surgery, open or laparoscopic methods, definition of SSI, size of groups, demographics (age; sex; body-mass index (BMI); smoking; American Society of Anesthesiologists physical status classification; history of hypertension, diabetes mellitus (DM), coronary artery disease, myocardial injury, chronic pulmonary diseases, cerebrovascular accidents; preoperative hemoglobin; and blood glucose levels), intraoperative and perioperative parameters (percentage of acute operations and transfusions; duration of surgery; and estimated blood loss), and outcomes.

The incidence of SSI was evaluated as the primary outcome, which was defined by either the CDC guidelines^6^ or the ASEPSIS scoring system^34^. Secondary outcomes were as follows: short-term mortality, myocardial injury, atelectasis, organ-space SSI, anastomotic leakage, pneumonia, and reoperation.

Two authors (JHK and WHK) independently evaluated the risk of bias of each selected randomized controlled trials (RCT) utilizing the Cochrane tool (RoB assessment tool version 2.0)^35^. Six domains were addressed: bias arising from the randomization process, bias due to deviations from intended interventions, bias due to missing outcome data, bias in the measurement of outcomes, and bias in the selection of reported outcomes. Risks were ranked as low, intermediate, or high. Overall risks of bias for each manuscript were reported as the highest risk among the five categories.

The quality of evidence for all study outcomes was assessed using the Grading of Recommendations Assessment, Development, and Evaluation (GRADE) methodology, which comprises five domains: risk of bias, inconsistency, indirectness, imprecision, and publication bias. Each outcome was rated as having very low, low, moderate, or high certainty of evidence.

### Data synthesis and analysis

Review Manager 5.4 software (RevMan, The Cochrane Collaboration, Oxford, United Kingdom) and STATA/SE version 14.0 (StataCorp, College Station, Texas, USA) were utilized for data synthesis and analysis. Binary data were analyzed via the Mantel–Haenszel method with a fixed-effects approach to calculate pooled risk ratios (RR) and 95% confidence intervals (CI), while the Inverse Variance method was applied to measure mean difference (MD) and 95% CI from continuous variables. Fixed effect model assumes one true effect size underlies all the studies in the meta-analysis. However, we also performed random effects approach to allow for the expected heterogeneity across the studies. The results of meta-analysis were depicted by forest plots for the primary outcome and hospital length of stay.

Subgroup analyses were conducted according to demographic data and surgical characteristics based on a priori analysis plan. Moderators were considered for analysis if they were known risk factors of SSI or if their clinical significance was acknowledged by all authors. Only those reported in a sufficient number of trials (at least 18) were ultimately selected, including mean BMI, percentage of patients with DM, urgency of surgery, type of surgery, carrier gas, durationof postoperative oxygenation supplementation, and percentage of current smokers. Continuous data such as mean BMI and duration of oxygen supplementation were converted into dichotomous categories.

Meta-regression was performed to assess the relationship between continuous covariates and our primary outcome. We applied criteria identical as mentioned above, resulting in age, percentage of males, mean BMI, percentage of patients with DM, duration of surgery, and percentage of smokers. Results are reported in bubble plots and corresponding *p* values. Publication bias was primarily assessed with funnel plots. Egger’s linear regression test was additionally performed for outcomes reported in 10 or more trials.

We performed trial sequential analysis (TSA) with TSA Viewer (Version 0.9.5.10 Beta, Copenhagen Trial Unit, 2016, Copenhagen, Denmark) for SSI, length of hospital stay, short-term mortality, and myocardial injury. It conducts a cumulative meta-analysis, manifested as a Z curve of the pooled observed effect, that reduces the risk of false-positive results from repetitive testing. We used a power of 80% and a 5% alpha error to calculate the required information size, which signifies the threshold for which the effect of the intervention may be confirmed or rejected. A conventional boundary denoting statistical significance (p < 0.05) and the trial sequential boundary (O’Brien-Fleming significance boundary) were also created. Overall, the course of the z-curve in relation to these borders helped us estimate when the effect will be large enough for future studies to be unnecessary. We used a 20% relative risk reduction (RRR) for binary outcomes, except for short-term mortality for which we used 10% RRR considering its clinical importance. For the continuous outcome (length of hospital stay), a mean difference of 0.5 was used.

A sensitivity analysis applying a random-effects approach was carried out to dismiss concerns regarding disparities among operation types and study designs, which may have breached assumptions necessary to undertake a fixed-effects approach. Another sensitivity analysis was done to compare trials with different definitions for SSI, namely per CDC guidelines, per ASEPSIS scoring system, and others. Heterogeneity was represented as the I^2^ statistic. We examined potential sources of heterogeneity via subgroup analyses and meta-regression.

## Results

**Figure 1:**
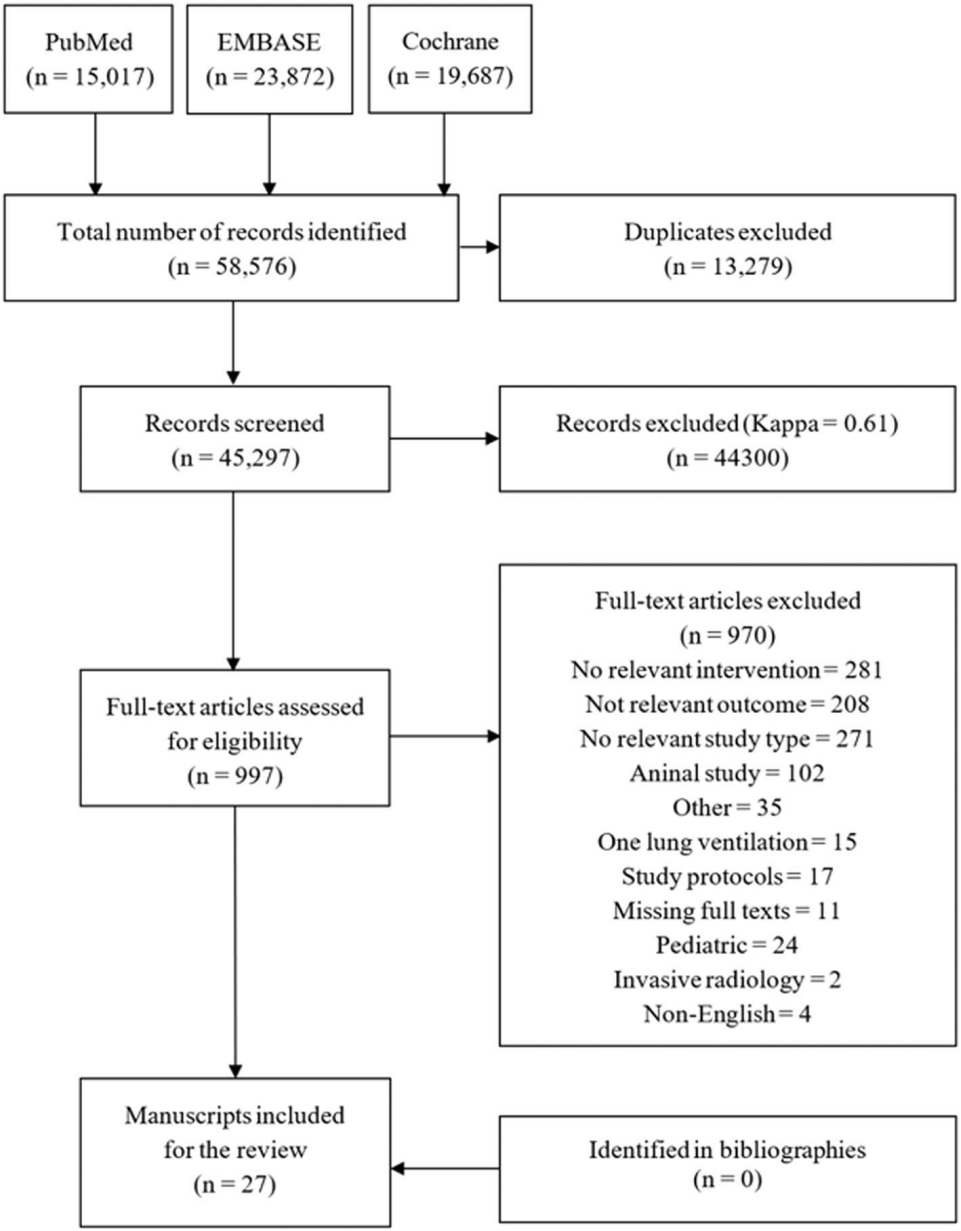
Study flow diagram.

We identified a total of 58,576 manuscripts from the initial search of which 13,279 duplicates were removed. After excluding 44,300 irrelevant studies, full texts of the remaining 997 studies were further inspected for eligibility. A total of 970 articles were removed for the reasons shown in Fig. 1. After evaluating trials additionally identified in references (n = 0), a sum of 27 original records comprising 15,977 patients was included in the final analysis.

The trials represented 25 parallel RCTs^31–33,36–57^, one cross-over RCT^58^, and one case–control study^59^. All but two^39,58^ were double-blinded trials. A total of 19 trials were conducted at a single center^31,36–39,41,45–48,50–54,56–59^, while 8 collected data from multiple centers^32,33,40,42–44,49,55^. Most of the trials compared a perioperative FiO_2_ of 80% to 30%, except three; one trial used 40% FiO_2_^46^, another used 35%^50^, and the other used 33% FiO_2_^47^ for the control arm (Table 1). Sample size of the trials ranged between 38 to 5749 (median 239), with 9 trials exceeding 500 patients (Table 1). Additional demographic and surgical characteristics are provided in Table 1 and Supplemental Table S1. The distribution of study outcomes across all included studies is shown in Supplemental Table S2.

**Table 1:** Baseline characteristics of the included trials.

Study ID	Sample size (n)		Age (year)		Sex (male/female, n)		FiO_2_ (%)		Type of anesthesia	Type of surgery
	High FiO_2_	Low FiO_2_	High FiO_2_	Low FiO_2_			High FiO_2_	Low FiO_2_		
Alvandipour 2019	40	40	58.4 (8.1)	59.2 (9.2)	26/14	23/17	80	30	General	Colorectal
Belda 2005	148	143	64.2 (11.8)	62.3 (12.5)	71/77	91/52	80	30	General	Colorectal
Bickel 2011	107	103	28.5 (12.3)	27.6 (10.8)	80/27	73/30	80	30	General	Appendectomy
Chen 2013	30	30	62 (12)	60 (15)	18/12	17/13	80	30	General	Colorectal
Duggal 2013	416	415	29.2 (5.6)	29.5 (5.8)	0	0	80	30	Regional	Caesarean section
Fariba 2016	61	61	29.7 (5.4)	29.3 (4.6)	0	0	80	30	Spinal	Caesarean section
Ferrando 2020	362	355	64.2 (12.8)	63.9 (13.9)	215/147	231/124	80	30	General or regional	Abdominal
Gardella 2008	69	74	31 [19–46]	28 [16–47]	0	0	80	30	Regional	Caesarean section
Greif 2000	250	250	57 (15)	57 (15)	143/107	137/113	80	30	General	Colorectal
Holse 2022	297	296	72 (9)	72 (9)	177/120	172/124	80	30	General	Noncardiac
Kurz 2015	285	270	54 (16)	51 (17)	144/135	134/132	80	30	Combined general and regional	Colorectal
Kurz 2018	2896	2853	52 (17)	52 (17)	1387/1509	1358/1495	80	30	Spinal or epidural	Major intestinal surgeries
Li 2020	126	125	54 (14)	53 (13)	75/51	71/55	80	30	General	Abdominal
Lin 2021	316	314	71.5 (3.9)	71.4 (2.8)	149/167	144/170	80	40	General	Gastric and colorectal
Mayank 2019	47	47	57.0 (12.9)	53.4 (14.5)	28/19	30/17	80	33	General	Colorectal
Mayzler 2005	19	19	67 (10)	69 (9)	10/9	12/7	80	30	General	Colorectal
Meyhoff 2009	685	701	64 (27–85)*	64 (34–84)*	288/406	297/409	80	30	General	Abdominal
Myles 2007	997	1015	55.8 (17)	54.6 (16)	533/444	520/495	80	30	General	Abdominal and gynecologic
Pryor 2004	80	80	54 (16)	57 (15)	34/45	34/46	80	35	General	Abdominal
Reiterer 2021	128	130	74 (70–78)	74 (70–78)	81/47	92/38	80	30	General	Abdominal
Schietroma 2013	86	85	68.4 [51–84]	67.8 [48–82]	50/36	48/37	80	30	General	Upper gastrointestinal
Schietroma 2016a	42	43	71.4 [55–92]	68.6 [49–86]	23/19	24/19	80	30	General	Colorectal
Schietroma 2016b	119	120	58.3 [35–80]	57.8 [30–82]	53/66	52/68	80	30	General	Perforated peptic ulcer
Wadhwa 2014	202	198	45 (12)	43 (12)	49/153	36/162	80	30	General	Roux-en-Y gastric bypass
Wasnik 2015	32	32	27.2 (10.5)	28.6 (12.2)	22/10	18/14	80	30	General	Appendectomy
Williams 2013	77	83	24.6	24.9	0	0	80	30	General	Caesarean section
Yerra 2021	93	85	40.2 (15.6)	39.1 (16.2)	69/24	64/21	80	30	General and spinal	Abdominal

Data are presented as mean (SD), median (1st–3rd quartile), or median [range].

FiO_2_ = inspired oxygen concentration, NR = not reported.

*5–95% percentile. General anesthesia was assumed when sevoflurane or isoflurane were used, or based on standard technique for the type of surgery in question.

Most studies showed an overall intermediate risk of bias, except for two determined to be at low risk^41,49^, and two at high risk^39,56^ (Supplemental Figure S1). GRADE approach showed that the quality of evidence of our study outcomes is from moderate to very low (Supplemental Table S3).

**Figure 2:**
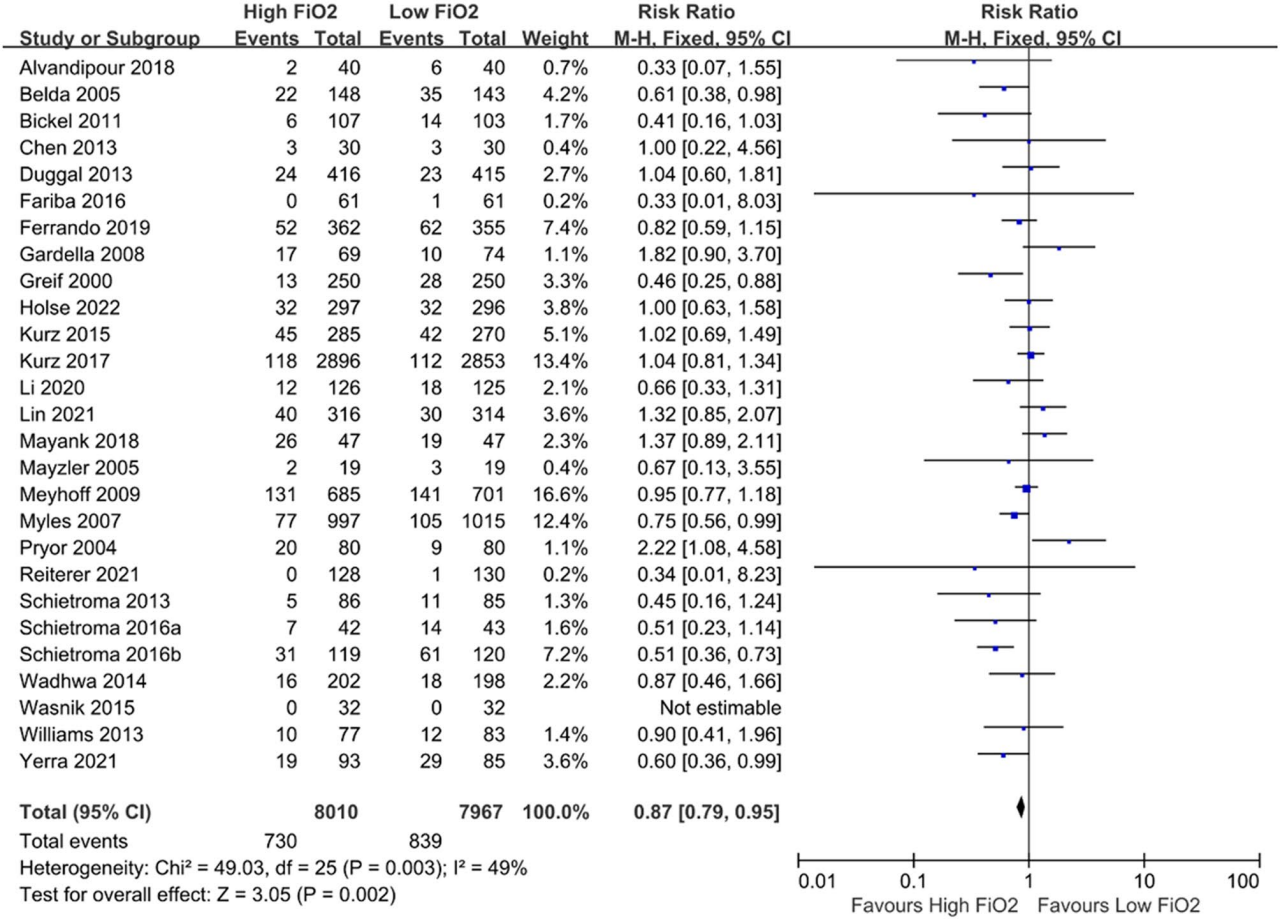
Forest plot of comparison between high FiO_2_ vs. low FiO_2_: surgical site infection. FiO_2_ = fraction of inspired oxygen.

As the inclusion criteria mandated the reporting of this outcome, all included 27 trials addressed the incidence of surgical site infection. Of these, 11 trials followed up with the patients for two weeks^31,33,36,38,39,41,42,49,50,53,54^ and another 11 trials for a month^32,37,40,43–45,47,48,51,58,59^. The remaining studies had various follow-up periods or did not specify the length of follow-up. Overall, the use of high FiO_2_ significantly reduced SSI (RR: 0.87; 95% CI: 0.79, 0.95; *p* = 0.002, I^2^ = 49%) (Fig. 2). The corresponding funnel plot does not suggest publication bias upon visual inspection (Supplemental Figure S2). The Egger test reinforces this assertion as no small-study effect was found (*p* = 0.773).

Twelve trials reported on the length of hospital stay for a total of 3703 patients^33,36,37,39–42,44–46,50,56^. There was no significant difference in the length of hospital stay between the two groups (MD: −0.06; 95% CI: −0.19, 0.08; *p* = 0.43, I^2^ = 66%) (Supplemental Figure S3). The funnel plot did not reveal any sign of publication bias, nor did the Egger test show a small-study effect (p = 0.645).

Data on mortality were available from thirteen trials involving 12,991 patients^32,40,42,43,45,46,49,51–55,58^. While short-term mortality of less than or equal to 60 days was reported in twelve trials^32,40,42,45,49,51–55,58^, two trials provided long-term mortality of six months^40,46^. The follow-up period for short-term mortality was 30 days for the overwhelming majority, save for two trials that tracked mortality for 60 days^55^ and 15 days^42^ each, and two trials that did not disclose the duration of follow-up^52,56^. No significant difference in short-term mortality was found between the two groups (RR: 1.04; 05% CI: 0.76, 1.43; *p* = 0.79, I^2^ = 34%) (Table 2). While the funnel plot was unremarkable, the Egger test warned of a small-study effect (*p* = 0.020).

**Table 2:** Summary of the results of secondary outcomes.

Outcomes	Events, High FiO_2_, n/N	Events, Low FiO_2_, n/N	Risk ratio (95% CI)	*p* value	I^2^
Short-term mortality^32,40,42,43,45,49,51–55,58^	79/6071	76/6051	1.04 (0.76, 1.43)	0.79	34
Myocardial injury^32,37,40,43,45,46,51,53,54^	108/2417	121/2428	0.90 (0.71, 1.13)	0.36	0
Atelectasis^31,32,40,45,46,49,59^	326/2611	296/2643	1.11 (0.96, 1.28)	0.16	88
Organ-space SSI^37,44,47,49^	42/1047	50/1048	0.85 (0.57, 1.27)	0.43	0
Anastomotic leakage^48,49,54,58,59^	61/4146	88/3741	0.59 (0.43, 0.79)	0.0006	83
Pneumonia^31,32,37,43,46,49^	106/2410	121/2449	0.89 (0.69, 1.14)	0.35	46
Reoperation^46,49–52,54^	128/1337	129/1353	1.01 (0.81, 1.27)	0.92	32

Outcome	High FiO_2_	Low FiO_2_	Mean difference (95% CI)	*p* value	I^2^
Length of hospital stay^33,36,37,39–42,44–46,50,56^			−0.06 (−0.19, 0.08)	0.43	66

FiO_2_ = inspired oxygen concentration, 95% CI = 95% confidence interval.

Eight studies of a total of 4845 patients reported the incidence of myocardial injury^32,37,40,43,45,46,51,53^. Two trials^43,51^ employed a standardized definition based on elevated troponin values, while the remaining six provided data on solely myocardial infarction. No notable difference was shown between the groups (RR: 0.90, 95% CI: 0.71, 1.13;* p* = 0.36, I^2^ = 0%) (Table 2). The funnel plot did not indicate any publication bias.

Regarding the incidence of atelectasis, an analysis of seven studies^31,32,40,45,46,49,59^ consisting of 5254 patients did not indicate a significant difference between groups (RR: 1.11; 95% CI: 0.96, 1.28; *p* = 0.16) (Table 2). Heterogeneity was very severe (I^2^ = 88%). The funnel plot was well-balanced.

Regarding organ-space SSI, six trials^31,37,44,47,49,58^ including a sum of 2095 patients reported data on the subcategories of SSI, namely superficial, deep, and organ-space. Among these, one trial^58^ documented a combination of deep and organ-space SSI, and another only noted deep SSI^31^. Following our decision to assess the incidence of organ-space SSI as a secondary outcome, these two trials were not included in the final analysis. There was no remarkable difference between the two groups (RR: 0.85; 95% CI: 0.57, 1.27, p = 0.43, I^2^ = 0%) (Table 2). The funnel plot did not suggest publication bias.

Six studies of a sum of 7887 patients demonstrated a decrease in anastomotic leakage incidence in the high FiO_2_ arm (RR: 0.59; 95% CI: 0.43, 0.79; *p* = 0.0006, I^2^ = 83%) (Table 2)^48,49,52,54,58,59^ Six trials documented data on pneumonia for 4859 patients^31,32,37,43,46,49^. The pooled estimate did not point out a significant difference between groups (RR: 0.89; 95% CI: 0.69, 1.14; *p* = 0.35, I^2^ = 46%). The funnel plot was not indicative of publication bias.

Reoperation data were available from six trials for a sum of 2690 patients^46,49–52,54^. High versus low FiO_2_ did not result in a meaningful reduction in reoperation rates (RR: 1.01; 95% CI: 0.81, 1.27; p = 0.92, I^2^ = 32) (Table 2). No publication bias was discerned from observing the funnel plot.

We further performed subgroup analyses on 9 major demographic and clinical characteristics for the primary outcome (Table 3). High FiO_2_ resulted in a significant decrease in SSI incidence for trials where the mean BMI was less than 30 (RR: 0.86; 95% CI: 0.75, 0.98; *p* = 0.03; I^2^ = 57%), while insignificant in more obese populations. SSI was also reduced in the subgroup where patients with diabetes mellitus constituted less than 20% of the entire population (RR: 0.86; 95% CI: 0.78, 0.95; *p* = 0.004; I^2^ = 56%), while otherwise not. The beneficial effect of high FiO_2_ was apparent in acute surgeries (RR: 0.54; 95% CI: 0.35, 0.84; *p* = 0.006; I^2^ = 0%) whereas unclear in trials composed of elective surgeries exclusively. FiO_2_ levels did not have a significant effect on SSI for Caesarean sections (RR: 1.15; 95% CI: 0.79, 1.67; *p* = 0.47; I^2^ = 0%) while a higher level was favorable for colorectal surgeries (RR: 0.78; 95% CI: 0.63, 0.97; *p* = 0.02; I^2^ = 51%) and other abdominal procedures (RR: 0.87; 95% CI: 0.78, 0.97; *p* = 0.01; I^2^ = 57%). Trials that used air as carrier gas resulted in SSI decrease for higher FiO_2_ (RR: 0.79; 95% CI: 0.69, 0.91;* p* = 0.001; I^2^ = 60) but not trials that used nitrogen or nitrogen oxide. The intervention reduced SSI in trials that supplied oxygen for at least six hours postoperatively (RR: 0.68; 95% CI: 0.56, 0.83; *p* = 0.0001; I^2^ = 46%); no such effect was shown in trials that supplied oxygen for shorter durations. A subgroup analysis on the percentage of smokers among patients did not yield significant results for either arm.

**Table 3:** Summary of the results of subgroup analysis for surgical site infection.

Covariates	Categories	Events, High FiO_2_, n/N	Events, Low FiO_2_, n/N	Risk ratio (95% CI)	*p *value	I^2^
N/A	Total	730/8010	839/7967	0.87 (0.79, 0.95)	0.002	49
Body-mass index (BMI)	BMI ≥ 30^38,41,55,57^	67/764	63/770	1.09 (0.78, 1.50)	0.62	0
	BMI < 30^33,39,40,44–54,58,59^	473/5124	534/5072	0.86 (0.75, 0.98)	0.03	57
Diabetes mellitus (DM)	DM ≥ 20%^37,43,46,51^	75/771	66/770	1.13 (0.83, 1.54)	0.44	0
	DM < 20%^4,31–33,36,38–41,44,47–50,52–54,56–59^	614/6661	709/6624	0.86 (0.78, 0.95)	0.004	56
Urgency of surgery	Acute^31,36^	25/200	43/188	0.54 (0.35, 0.84)	0.006	0
	Elective^33,37,39,41,42,44–48,51–53,55,57^	218/1886	244/1872	0.89 (0.75, 1.05)	0.15	38
Type of surgery	Caesarean section^38,39,41,57^	51/623	46/633	1.15 (0.79, 1.67)	0.47	0
	Colorectal surgery^33,37,42,44,47,48,54,59^	120/861	150/842	0.78 (0.63, 0.97)	0.02	51
	Other abdominal surgery^31,32,36,40,43,45,46,49–53,55,56,58^	538/6526	615/6492	0.87 (0.78, 0.97)	0.01	57
Carrier gas	Air^31,33,41,43,45,49,52–54^	276/1665	350/1672	0.79 (0.69, 0.91)	0.001	60
	N_2_ or N_2_0^32,36,37,42,44,47,48,50,59^	194/1855	229/1854	0.84 (0.71, 1.01)	0.06	61
Time of oxygen supplementation	≥ 6 h postoperative^33,37,39,45,47,52–55^	122/861	179/852	0.68 (0.56, 0.83)	0.0001	46
	< 6 h postoperative^31,32,36,38,40–44,46,48–51,56–59^	604/7079	655/7050	0.92 (0.83, 1.02)	0.12	44
Current smoker	Smokers ≥ 20%^32,33,43,44,46,47,49,57,59^	385/2892	422/2909	0.92 (0.81, 1.04)	0.18	38
	Smokers < 20%^31,37,38,40,42,45,50,55,58^	277/4455	301/4391	0.90 (0.77, 1.05)	0.20	47

FiO_2_ = inspired oxygen concentration, 95% CI = 95% confidence interval.

Meta-regression revealed no significant interaction between SSI with any covariates, namely age (*p* = 0.73), sex (*p* = 0.06), BMI (*p* = 0.99), DM (*p* = 0.244), duration of surgery (*p* = 0.295), and smoking (*p* = 0.696) (Supplemental Figure S4).

**Figure 3:**
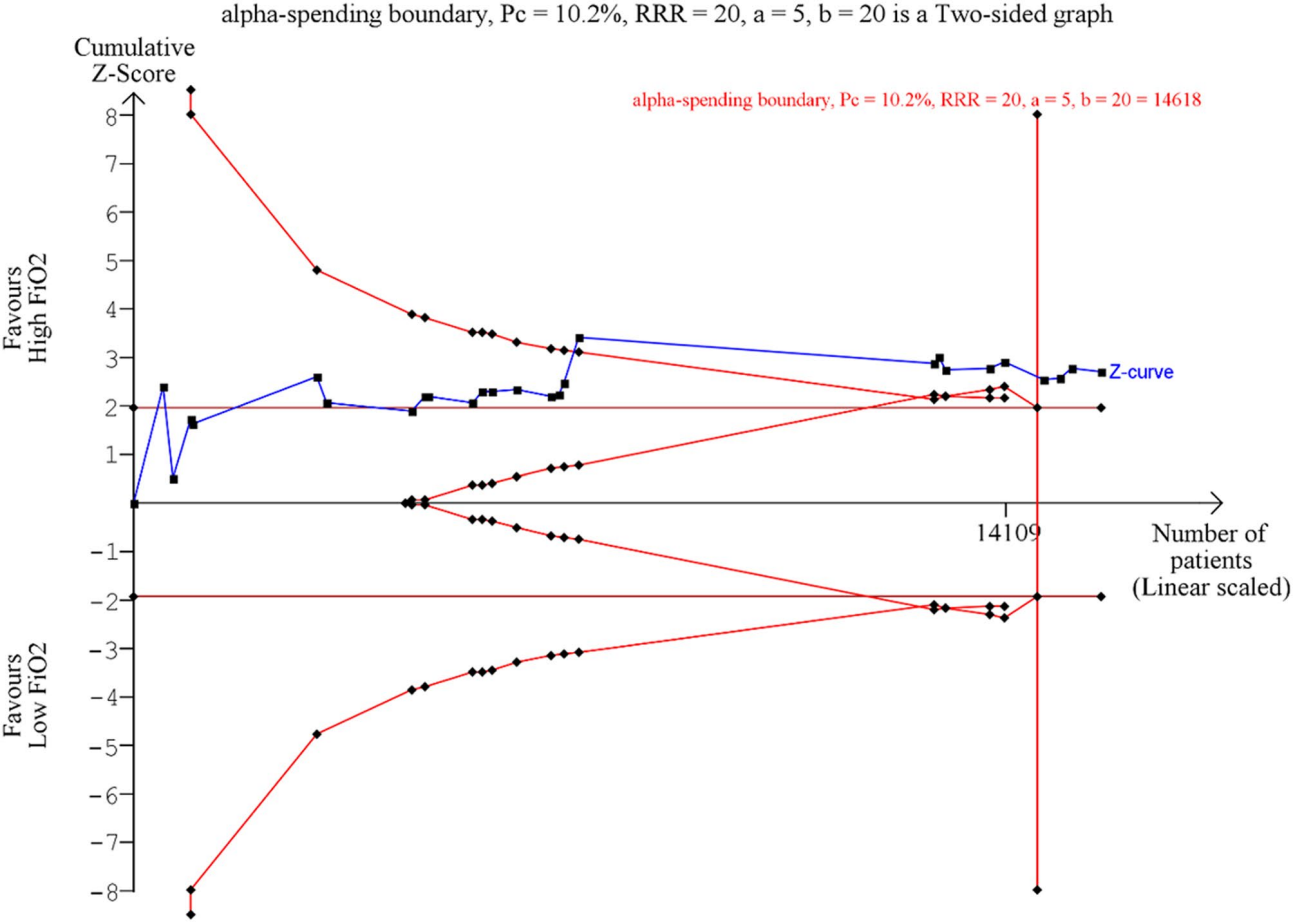
Trial sequential analysis for surgical site infection. Pc = Probability in the control group, RRR = relative risk reduction, a = alpha error, b = beta-error. The blue line means the cumulative z-score curve. The boundaries favoring high or low FiO2 or area of futility are shown in red lines.

TSA for SSI revealed that the z-curve surpassed both the conventional boundary and the O’Brien Fleming significance boundary while also exceeding the required sample size (Fig. 3). This indicates that further trials are unlikely to alter our conclusion that high FiO_2_ decreases SSI in abdominal surgeries. On the other hand, the z-curve for the length of hospital stay passed the required sample size within the area of futility (Supplemental Figure S5). The curves for short-term mortality (Supplemental Figure S6), myocardial injury (Supplemental Figure S7), and atelectasis (Supplemental Figure S8) remain within the conventional boundary and have yet to reach their respective required sample sizes, pointing out a need for additional data.

A sensitivity analysis applying the random-effects model did not change the effect direction or statistical significance for all outcomes (Supplemental Table S4). A second sensitivity analysis for SSI incidence was performed on the heterogeneous definitions of SSI. Differences between the two groups remained significant for trials defining SSI per ASEPSIS score (RR: 0.43; 95% CI: 0.26, 0.70; *p* = 0.0007; I^2^ = 0%) and CDC guidelines (RR: 0.87; 95% CI: 0.79, 0.96; *p* = 0.006; I^2^ = 34%) alike. Thirdly, a sensitivity analysis of trials that strictly conformed with WHO recommendations (i.e. trials on patients 18 years or older under general anesthesia)^32,33,37,42–55,59^ yielded results consistent with the initial analysis (RR: 0.84; 95% CI: 0.75, 0.94; *p* = 0.002; I^2^ = 54%). No significant effect was found in trials deviating from the guidelines^36,38,39^ (RR: 0.94; 95% CI: 0.79, 1.12; *p* = 0.52, I^2^ = 35%). Fourthly, the trials by Schietroma et al. should be dealt with cautiously due to retractions among the author’s other manuscripts^60^. We performed a sensitivity analysis removing the works by Schietroma et al. yielded no significant effect of FiO_2_ (Supplemental Table S5)^52–54^.

## Discussion

Our meta-analysis demonstrated a potential benefit of a FiO_2_ of 80% compared to 30% or lower in reducing SSI in abdominal surgeries. TSA revealed that the required sample size was reached and the cumulative z-curve crossed the trial sequential boundary of the preference for high FiO_2_. A sensitivity analysis of the definition of SSI did not alter the results. However, the low quality of evidence still prevents a firm conclusion.

Whilst evidence for WHO guidelines are disputed, the beneficial effect of high FiO_2_ on SSI reduction continued to be found in recent studies, albeit in limited populations. It has been previously demonstrated for a subgroup of intubated patients^23^, or trials on a majority of emergency operations^21^, and when a stringent value of 80% versus 30% oxygen was applied^21^. This finding was also supported by a previous meta-analysis when inadequately or ambiguously blinded trials were excluded^61^.

Wound infections are usually established in a “decisive period” of several hours in the postoperative period^9^, during which host defense vigorously functions to remove pathogens, starting with neutrophils. Their key mechanism is the generation of antimicrobial reactive oxidant species^9,42^. In vitro studies have shown that neutrophil oxygen consumption and hence its production of oxidants are hampered at low oxygen tension^8^, which is alleviated by the administration of high levels of oxygen^62^. Therefore, it has been hypothesized that high FiO_2_ activates the antimicrobial mechanism of neutrophils and thereby decreases SSI. This theory was true in an animal study which showed that neutrophils in the 80% FiO_2_ group were more activated than those in the 30% group, although no variance was found in infiltration amounts^63^. Nonetheless, clinical trials have yielded mixed results due to various confounding factors of the clinical practice. However, the significant result of our subgroup analysis of supplied oxygen for at least postoperative six hours supports the importance of the decisive period.

Our results contrast with the cumulative outcomes of recent meta-analyses that found no significant beneficial or harmful effect of high FiO_2_^21–23,61,64,65^. The different result of our meta-analysis is primarily due to the inclusion of only abdominal surgeries while previous meta-analyses included any types of surgeries. Three previous meta-analyses included randomized trials of non-cardiac or any type of surgeries and reported no significant results for SSI^21,61,65^. The other meta-analyses analyzed mortality, length of hospital stay, and the incidence of cardiovascular or respiratory complication but reported no significant difference^22,23,64^, which was consistent with our results. The significant results of our meta-analysis for SSI may be, in part, attributed to certain characteristics specific to abdominal surgery. One possible explanation is a marked variance in pathogens. While *Staphylococcus aureus* is the most common microorganism causing SSI^66^, *Escherichia coli* is the most predominant in SSI following abdominal surgery^67^. *S. aureus* is a notorious foe to neutrophils, armed with mechanisms for evasion such as chemotaxis inhibitory protein^68^ and extracellular fibrinogen binding proteins that block complement activation^69^. *E. coli* is armed with other defense mechanisms such as lipopolysaccharides. As such, discrepancies in bacterial characteristics interact with neutrophil activity differently; perhaps those that infest abdominal wounds are more susceptible to neutrophils, which, in turn, may enhance the effect of high FiO_2_.

The high FiO_2_ was also associated with a significantly lower incidence of anastomotic leakage. Ischemia in surrounding tissue is essential for anastomotic leakage development, leading to delayed wound healing, necrosis, and dehiscence^70^. Application of high FiO_2_ increases tissue oxygen levels and may thereby prevent the formation of anastomotic leakage. No association was found for organ-space SSI; there is yet no evidence for a beneficial effect of oxygen on severe SSI. Previous studies and ours alike showed no association between FiO_2_ and poor clinical outcomes including mortality, myocardial injury, reoperation, pneumonia, and in length of hospital stay^21–23^.

However, the adverse effect of high FiO_2_ should be acknowledged. Given that one hundred percent oxygen is known to induce absorption atelectasis even if administered for a short time^71^ and that another meta-analysis demonstrated detrimental effects of high FiO_2_ on oxygen parameters and severity of atelectasis^72^, concerns about pulmonary function cannot be discarded. Atelectasis is not causative of postoperative pneumonia^73^ and must be dealt with as an independent outcome.

TSA for SSI revealed that we have reached the required information size and the O’Brien-Fleming boundary had been crossed. Nonetheless, the quality of evidence was judged low; robust evidence is still lacking, and our results are subject to change. Secondary adverse outcomes of high FiO_2_ have yet to reach the required information size.

A subgroup analysis showed that high FiO_2_ is beneficial only when the mean BMI was below 30. This result lacks sufficient power as revealed by TSA and is contrary to our expectation; obesity is a known risk factor for surgical site infection^74^, especially for colorectal surgery^75^. Hypoperfusion of adipose tissue in obese patients delays wound healing and forms dead space, predisposing patients to SSI^76^. Additionally, poor tissue oxygenation makes it difficult for prophylactic antibiotics to reach sufficient concentrations.

We found high FiO_2_ to be beneficial in emergency operations, consistent with the subgroup analysis of a previous meta-analysis^21^ yet contrasting with another study on acutely ill adults^16^ and a third inconclusive study^77^. These discrepancies may be due to insufficient power and heterogeneity in included surgery types. The previous meta-analysis^21^ was based on three trials of 509 patients and our analysis was on only two trials with 388 patients. Studies on acute appendicitis took up the majority with a short operation time duration and a predominantly laparoscopic approach.

Subgroup analysis comparing nitrous oxide to air showed that FiO_2_ decreased SSI in only the latter. While a previous meta-analysis claimed that usage of nitrous oxide does not significantly alter SSI rates^78^, its assertion may lack power as it was based on six trials with high heterogeneity. A randomized trial published afterward also found no association between N_2_O and SSI^79^. We believe that N_2_O may potentially serve as a confounding factor; it is known to inhibit methionine production, which leads to a reduction in protein expression which in turn deters the healing process^80^. It is also known to depress chemotactic migration^81^ and inhibit methionine synthase^82^.

Subgroup analysis revealed that the beneficial effect of high FiO_2_ on SSI was consistent to a subgroup of colorectal surgeries and other abdominal surgeries, but not to trials of Cesarean sections. Caesarean sections are commonly operated under regional anesthesia, which was the case for all trials included in our analysis. It is also a relatively safe procedure with a low SSI rate, hence the effect of FiO_2_ may have been statistically trivial even if favorable.

High FiO_2_ appeared to be useful only when the duration of exposure was 6 h or longer. This starkly contrasts with the current WHO guidelines, which advise oxygen administration for 2–6 h postoperatively^5^. The suggested duration is not based on physiological evidence and is rather attributable to additional factors such as resource use. As far as we know, the optimal duration of exposure has not been meticulously studied to date. The decisive period for oxygen to benefit a patient is unknown; and though a need for a direct comparison between exposure duration has been suggested^33^, we found no trials delving into this issue. Further trials comparing the effect of oxygenation for different durations are required to better understand the effect of oxygen on SSI reduction.

Our meta-analysis provided timely analysis, including recently published studies^31,43^ and a trial previously left out for unknown causes^55^, as well as re-incorporating studies that have been omitted from conservative meta-analyses due to concerns of authors with retracted articles^52–54^. Despite several previous meta-analyses on broader classes of surgery, an inspection of abdominal surgery has not been conducted. It has been analyzed as a subgroup analysis with insignificant results^21^. Including recently published trials and trials with regional anesthesia as well have resulted in a contrasting conclusion. We also performed a subgroup analysis on diabetes mellitus and BMI, well-known risk factors of SSI. Our findings that diabetes and obesity may hinder or counterbalance the positive effects of oxygen may contribute to stratifying the intervention to relevant patients in future operations. While concerns have been raised on the vast range of postoperative oxygenation duration^16^, no analysis has been done on the topic; we found a significant benefit of oxygenation administered for at least 6 h. Heterogeneous definitions of the endpoint were also brought up as a source of potential imprecision, yet our sensitivity analysis showed that trials on CDC or ASEPSIS definitions benefited from the intervention alike. A final sensitivity analysis including only the population under general anesthesia with tracheal intubation as indicated by WHO guidelines revealed that high FiO_2_ does indeed reduce SSI. This finding effectively diminishes the risks of imprecision arising from our attempt to include more studies than previous reviews. Meta-regression found that age, mean BMI, percentage of patients with DM, and percentage of smokers did not significantly alter our results. Male sex is a disputed risk factor; a recently published multicenter study found its effect non-significant^83^.

There are several important limitations in our study. Firstly, the quality of evidence is low. Only two^41,49^ included trials were deemed at low risk of bias, while two trials judged to be at high risk^39,56^ impose concerns on the possible bias. We rated high-risk trials due to the concerns with deviations from intended interventions^39^ and outcome measurement^56^. Regarding ethical consideration, the studies by Schietroma et al. should be dealt with caution due to questionable methodology based on some retractions^60^. Some of other studies by Schietroma et al. were retracted, although the studies included in our analyses were not retracted or are under investigation^60^. Our sensitivity analysis removing the works of Schietroma yielded no significant effect of FiO_2_^52–54^.

Secondly, heterogeneity among data is also a downfall. The I^2^ value for the main analysis and notable subgroup analyses was high. We attempted to explore major potential sources of heterogeneity with subgroup analyses and meta-regression; unresolved factors may include variations in anesthetic regimes and protocols of prophylactic antibiotics. Aspects of the study population such as comorbidities or selectioncriteria may also have played a role. Study settings and the baseline quality of performance of each research center were also discrepant. As surgical and anesthetic techniques have evolved, older trials may be outdated; the year of inclusion showed indeed a significant influence^21^. Variance in sample size is another potential source of heterogeneity, as was found in the aforementioned study.

In conclusion, this meta-analysis found that high FiO_2_ reduced the incidence of SSI and anastomotic leakage after abdominal surgery, a viewpoint distinct from the current consensus that perioperative high FiO_2_ does not benefit patients. This difference may be attributable to our focus on solely abdominal operations. Taking together the variance in subgroups, we suggest that the administration of high FiO_2_ should be individualized based on patient and surgery characteristics rather than being standardized. Based on our significant subgroup analyses for colorectal surgery and other abdominal surgeries but not for Caesarean section, further studies for these specific types of surgeries are required. The TSA for SSI revealed that the cumulative analysis crossed the trial sequential boundary while the required information size was reached. However, our results of the meta-analysis and TSA should be cautiously interpreted as the quality of evidence was judged moderate to very low for all outcomes. Further large trials focusing on high-risk surgeries, and a direct comparison of oxygenation duration are required to explore the true effects of this intervention which underlies countless operations.

### Supplementary Information

Supplementary Information.

## Data Availability

All other data is available in the Supplementary Information files. Any further information including all data used in our analyses are available upon request from the corresponding author.

## Abbreviations

BMI: Body-mass index
CDC: Centers for Disease Control and Prevention
CI: Confidence interval
DM: Diabetes mellitus
FiO_2_: Fraction of inspired oxygen
GRADE: Grading of Recommendations Assessment, Development, and Evaluation
MD: Mean difference
RCT: Randomized controlled trials
RR: Relative risk
RRR: Relative risk reduction
SSI: Surgical site infection
TSA: Trial sequential analysis
WHO: World Health Organization
