# Supplementary material for: The effect of high perioperative inspiratory oxygen fraction for abdominal surgery on surgical site infection: a systematic review and meta-analysis
Source: Sci Rep. 2023 Sep 20;13:15599. doi: 10.1038/s41598-023-41300-4 (PMC10511429; doi:10.1038/s41598-023-41300-4)

**The effect of high perioperative inspiratory oxygen fraction for abdominal surgery on surgical site infection: a systematic review and meta-analysis: Supplemental materials**

**Supplemental Text S1**. Search strategy.

**Supplemental Text S2**. Inclusion and exclusion criteria.

**Supplemental Table S1**. Other baseline characteristics of the included trials.

**Supplemental Table S2**. Distribution of study outcomes across the included manuscripts.

**Supplemental Table S3**. Quality of evidence (GRADE approach).

**Supplemental Table S4**. Sensitivity analysis was performed (1) applying the random-effects model for all study outcomes, (2) according to the different definitions of surgical site infection, and (3) based on compliance with WHO guidelines.

**Supplemental Table S5**. Sensitivity analysis was performed by removing all trials by Schietroma et al.

**Supplemental Figure S1**. Risk of bias assessment of the included studies (version 2.0).

**Supplemental Figure S2**. Funnel plots of the following outcomes: A = surgical site infection, B = length of hospital stay, C = short-term mortality, D = myocardial injury, E = atelectasis.

**Supplemental Figure S3**. Forest plot of comparison between high FiO_2_ vs. low FiO_2_: length of hospital stay. FiO_2_ = fraction of inspired oxygen.

**Supplemental Figure S4**. Bubble plot for meta-regression of surgical site infection and covariates: A = age, B = percent of males, C = body-mass index, D = percent of patients with diabetes mellitus, E = duration of surgery, F = percent of current smokers.

**Supplemental Figure S5**. Trial sequential analysis for length of hospital stay.

**Supplemental Figure S6**. Trial sequential analysis for short-term mortality. Pc = Probability in the control group, RRR = relative risk reduction, a = alpha error, b = beta-error.

**Supplemental Figure S7**. Trial sequential analysis for myocardial injury. Pc = Probability in the control group, RRR = relative risk reduction, a = alpha error, b = beta-error.

**Supplemental Figure S8**. Trial sequential analysis for atelectasis. Pc = Probability in the control group, RRR = relative risk reduction, a = alpha error, b = beta-error.

**Supplemental Text S1**. Search strategies

**1. Medline**

(Anesthesia, General[Mesh] OR universal anesthesia[Title/Abstract] OR universal

anaesthesia[Title/Abstract] OR general anesthesia[Title/Abstract] OR general

anaesthesia[Title/Abstract] OR General Surgery[Mesh] OR surgery[Title/Abstract] OR

surgical[Title/Abstract] OR perioperative[Title/Abstract] OR “peri-operative”[Title/Abstract] OR intraoperative[Title/Abstract] OR “intra-operative”[Title/Abstract])

AND

(oxygen[Title/Abstract] OR FiO2[Title/Abstract] OR hyperoxygenation[Title/Abstract] OR

hyperbaric[Title/Abstract])

AND

(randomized controlled tri-al[Publication Type] OR controlled clinical trial[Publication Type] OR randomized[Title/Abstract] OR placebo[Title/Abstract] OR clinical trials as topic[Mesh:noexp] OR randomly[Title/Abstract] OR trial[Title]) NOT (animals[Mesh] NOT humans [Mesh]) AND (English[Language])

**2. Cocharane CENTRAL**

1. (Anesthesia, General OR universal anesthesia OR universal anaesthesia OR general anesthesia

OR general anaesthesia OR General Surgery OR surgery OR surgical OR perioperative OR perioperative OR intraoperative OR intra-operative)

2. (oxygen OR FiO2 OR hyperoxygenation OR hyperbaric)

3. (randomized controlled trial OR controlled clinical trial OR randomized OR placebo OR clinical trials

OR randomly OR trial)

4. #1 and #2 and #3

**Supplemental Text S2**. Inclusion and exclusion criteria.

1. Inclusion criteria

1) A randomized trial enrolling patients of 18 years or older undergoing any type of abdominal surgery under general or spinal anesthesia.

2) A randomized trial comparing perioperative administration of high-concentration oxygen (80% or more) versus a control group provided with standard concentration oxygen (30-40%).

3) A randomized trial on human patients provided in full-text English.

2. Exclusion criteria

1) A randomized trial enrolling pediatric patient (< 18 years)

2) A randomized trial with missing oxygen data

3) A randomized cross-over trials, where the cross-over occurred within individual patients.

**Supplemental Table S1**. Other baseline characteristics of the included trials.

| Study ID | Outcome definition | Postoperative  oxygenation (h) | Carrier gas | Body-mass index | | Diabetes mellitus (n) | | Hypertension (n) | | Current smoker (n) | |
| --- | --- | --- | --- | --- | --- | --- | --- | --- | --- | --- | --- |
|  |  |  |  | High FiO_2_ | Low FiO_2_ | High FiO_2_ | Low FiO_2_ | High FiO_2_ | Low FiO_2_ | High FiO_2_ | Low FiO_2_ |
| Alvandipour 2019 | ASEPSIS | 1 | nitrous oxide | 26.2 (8.1) | 25.9 (6.8) | 5 | 4 | 4 | 3 | 9 | 8 |
| Belda 2005 | CDC | 6 | air | 27.1 (4.5) | 26.5 (3.8) | 0 | 0 | NR | NR | 30 | 30 |
| Bickel 2011 | ASEPSIS | 2 | nitrogen | NR | NR | 0 | 1.4 | 10 | 5 | NR | NR |
| Chen 2013 | CDC | 24 | nitrogen | NR | NR | 13 | 10 | NR | NR | 2 | 3 |
| Duggal 2013 | CDC | 1 | NR | 34.9 (7.1) | 34.2 (6.7) | 91 | 87 | NR | NR | 12 | 14 |
| Fariba 2016 | ASEPSIS | 6 | NR | 27.5 (5) | 28 (4.5) | 0 | 0 | 0 | 0 | NR | NR |
| Ferrando 2020 | CDC | 3 | NR | 26.2 (3.8) | 26.4 (3.8) | 62 | 74 | 161 | 161 | 74 | 66 |
| Gardella 2008 | administration of antibiotics  or wound infection during 14 days | 2 | air | 32 [22-51] | 32 [20-49] | 0 | 0 | 16 | 31 | NR | NR |
| Greif 2000 | ASEPSIS | 2 | nitrogen | NR | NR | NR | NR | NR | NR | 60 | 73 |
| Holse 2022 | CDC | 2 | air | NR | NR | 63 | 55 | 209 | 208 | 234 | 217 |
| Kurz 2015 | CDC | 1 | nitrogen | 27 (6) | 27 (7) | 9 | 9 | NR | NR | 57 | 68 |
| Kurz 2018 | CDC | 0 | NR | 26 (22-30) | 26 (23-31) | 336 | 325 | 1022 | 1025 | 320 | 263 |
| Li 2020 | CDC | 24 | air | NR | NR | NR | NR | 19 | 11 | 18 | 21 |
| Lin 2021 | NR | 0 | NR | 22.76 (1.31) | 22.75 (1.3) | 72 | 63 | 108 | 123 | 73 | 74 |
| Mayank 2019 | CDC | 6 | nitrous oxide | 21.47 (1.45) | 21.23 (1.6) | 7 | 5 | 8 | 8 | 12 | 13 |
| Mayzler 2005 | Erythema with local pain  and drainage of fluid or purulent secretion | 2 | nitrogen | 25 (4) | 27 (5) | 0 | 0 | NR | NR | NR | NR |
| Meyhoff 2009 | CDC | 2 | air | 25 (18-35) † | 25 (19-35) † | 51 | 53 | 209 | 187 | 207 | 213 |
| Myles 2007 | CDC | 0 | nitrogen or  nitrous oxide | NR | NR | 137 | 140 | 322 | 356 | 184 | 234 |
| Pryor 2004 | self-defined* | 2 | nitrous oxide | 27.1 (6.7) | 25.1 (5.0) | 7 | 8 | 23 | 28 | 7 | 13 |
| Reiterer 2021 | NR | 2 | NR | 26.7 (24.0-30.7) | 25.8 (23.7-29.1) | 39 | 35 | 120 | 120 | NR | NR |
| Schietroma 2013 | graded as Franchi et al.† | 6 | air | 24.5 [18.1-36.2] | 23.8 [16.9-35.7] | 0 | 0 | NR | NR | NR | NR |
| Schietroma 2016a | CDC | 6 | air | 25.8 [18.4-36.2] | 24.4 [17.1-37.9] | 0 | 0 | NR | NR | NR | NR |
| Schietroma 2016b | CDC | 6 | air | 26.8 [18.6-40.1] | 24.9 [18.1-39.4] | 0 | 0 | NR | NR | NR | NR |
| Wadhwa 2014 | CDC | to the first  postoperative morning | NR | 46 (42-52) | 46 (42-54) | NR | NR | NR | NR | 32 | 25 |
| Wasnik 2015 | ASEPSIS | 2 | NR | NR | NR | 0 | 0 | NR | NR | NR | NR |
| Williams 2013 | CDC | 2 | NR | 34.9 | 36.9 | 11 | 5 | 14 | 16 | 17 | 15 |
| Yerra 2021 | CDC | 2 | air | NR | NR | 9 | 6 | 10 | 12 | 19 | 14 |

FiO_2_ = inspired oxygen concentration, NR = not reported, CDC = Centers for Disease Control and Prevention, ASEPSIS = Additional treatment, presence of Serous discharge, Erythema, Purulent exudate, Separation of the deep tissues, Isolation of bacteria, and the duration of inpatient Stay; a patient is concluded to have surgical site infection when the sum of scores for each parameter exceeds 20.

Postoperative oxygenation refers to the duration for which high or low levels of FiO_2_ was maintained after surgery ended.

*(1) The surgical team clearly documented a clinical assessment of SSI; (2) the infection precipitated a management action, such as the initiation or changing of antibiotics, opening of the wound, aspiration, drain placement, or further surgery; and (3) the clinical assessment was supported by the presence of at least 3 or the following objective criteria: (a) a white blood cell count higher than 11,000/ μL; (b) temperature higher than 38.5 ; (c) radiological evidence of infection; (d) extrusion of pus from the wound; (e) positive culture result from the infected site; and (f) documentation of wound erythema and induration on physical examination that resolved with treatment of infection.^51^

†Grade from reference ^84^ Franchi M, Ghezzi F, Zanaboni F, Scarabelli C, Beretta P, Donadello N. Nonclosure of peritoneum at radical abdominal hysterectomy and pelvic node dissection: a randomized study. Obstet Gynecol 1997;90:622-7.

**Supplemental Table S1**. Other baseline characteristics of the included trials (continued).

| Study ID | Acute surgery (%) | | Duration of surgery (min) | |
| --- | --- | --- | --- | --- |
|  | High FiO_2_ | Low FiO_2_ | High FiO_2_ | Low FiO_2_ |
| Alvandipour 2019 | 30 | 35 | NR | NR |
| Belda 2005 | 0 | 0 | 161 (62) | 159 (61) |
| Bickel 2011 | 100 | 100 | 33.0 (10.6) | 32.8 (10.3) |
| Chen 2013 | 0 | 0 | NR | NR |
| Duggal 2013 | some | some | 104 [25.1] | 94 [22.7] |
| Fariba 2016 | 0 | 0 | 57.5 (14.2) | 60 (10.4) |
| Ferrando 2020 | NR | NR | 214.2 (93.3) | 209.2 (89.7) |
| Gardella 2008 | 0 | 0 | 48 [26-87] | 52 [20-141] |
| Greif 2000 | 0 | 0 | 3.1 (1.4) | 3.1 (1.4) |
| Holse 2022 | 10 | 8.8 | 127 (52-303) | 137 (54-316) |
| Kurz 2015 | 0 | 0 | 3.5 (1.5) | 3.5 (1.8) |
| Kurz 2018 | NR | NR | 4.0 (1.7) | 4.0 (1.7) |
| Li 2020 | 0 | 0 | 190 (154-245) | 200 (146-258) |
| Lin 2021 | 0 | 0 | 188.2 (12.7) | 188.7 (8.6) |
| Mayank 2019 | 0 | 0 | 233.5 (87.7) | 207.7 (103.5) |
| Mayzler 2005 | 0 | 0 | 140 (40) | 135 (40) |
| Meyhoff 2009 | 28 (overall) |  | 128 (38-310) | 132 (35-295) |
| Myles 2007 | 4 | 4 | 3.3 (2.0) | 3.3 (2.0) |
| Pryor 2004 | some | some | 233 (83) | 208 (91) |
| Reiterer 2021 | 0 | 0 | 194 (136-255) | 195 (128-286) |
| Schietroma 2013 | 0 | 0 | 175 [100-250] | 180 [180-270] |
| Schietroma 2016a | 0 | 0 | 200 [95-410] | 195 [100-385] |
| Schietroma 2016b | NR | NR | 63.2 [38-104] | 58.7 [33-102] |
| Wadhwa 2014 | 0 | 0 | 2.7 (2.1-3.2) | 2.6 (2.0-3.3) |
| Wasnik 2015 | NR | NR | 61.9 (10.6) | 61.4 (12.5) |
| Williams 2013 | 0 | 0 | 52.1 | 52.2 |
| Yerra 2021 | 100 | 100 | 160.8 (72.6) | 154.24 (67.9) |

FiO_2_ = inspired oxygen concentration, NR = not reported.

**Supplemental Table S2.** Distribution of study outcomes across the included manuscripts.

|  | Surgical site  infection | Anastomotic  leakage | Reoperation | Organ-space  infection | Short-term  mortality (30 days) | Atelectasis | Pneumonia | Myocardial injury | Length of  hospital  stay |
| --- | --- | --- | --- | --- | --- | --- | --- | --- | --- |
| Alvandipour 2019 | ✓ | ✓ |  |  |  | ✓ |  |  |  |
| Belda 2005 | ✓ |  |  |  |  |  |  |  | ✓ |
| Bickel 2011 | ✓ |  |  |  |  |  |  |  | ✓ |
| Chen 2013 | ✓ |  |  | ✓ |  |  | ✓ | ✓ | ✓ |
| Duggal 2013 | ✓ |  |  |  |  |  |  |  |  |
| Fariba 2016 | ✓ |  |  |  |  |  |  |  | ✓ |
| Ferrando 2020 | ✓ |  |  |  | ✓ | ✓ |  | ✓ | ✓ |
| Gardella 2008 | ✓ |  |  |  |  |  |  |  | ✓ |
| Greif 2000 | ✓ |  |  |  | ✓ |  |  |  | ✓ |
| Holse 2022 | ✓ |  |  |  | ✓ |  | ✓ | ✓ |  |
| Kurz 2015 | ✓ |  |  | ✓ |  |  |  |  | ✓ |
| Kurz 2018 | ✓ | ✓ |  | ✓ | ✓ |  |  |  |  |
| Li 2020 | ✓ |  |  |  | ✓ | ✓ |  | ✓ | ✓ |
| Lin 2021 | ✓ |  | ✓ |  |  | ✓ | ✓ | ✓ | ✓ |
| Mayank 2019 | ✓ |  |  | ✓ |  |  |  |  |  |
| Mayzler 2005 | ✓ | ✓ |  |  |  |  |  |  |  |
| Meyhoff 2009 | ✓ | ✓ | ✓ | ✓ | ✓ | ✓ | ✓ |  |  |
| Myles 2007 | ✓ |  |  |  | ✓ | ✓ | ✓ | ✓ |  |
| Pryor 2004 | ✓ |  | ✓ |  |  |  |  |  | ✓ |
| Reiterer 2021 | ✓ |  | ✓ | ✓ | ✓ |  |  | ✓ |  |
| Schietroma 2013 | ✓ | ✓ | ✓ |  | ✓ |  |  |  |  |
| Schietroma 2016a | ✓ | ✓ | ✓ |  | ✓ |  |  |  |  |
| Schietroma 2016b | ✓ |  |  |  |  |  |  | ✓ |  |
| Wadhwa 2014 | ✓ |  |  |  | ✓ |  |  |  |  |
| Wasnik 2015 | ✓ |  |  |  |  |  |  |  | ✓ |
| Williams 2013 | ✓ |  |  |  |  |  |  |  |  |
| Yerra 2021 | ✓ |  |  | ✓ |  | ✓ | ✓ |  |  |

✓ indicates the presence of the corresponding outcome.

**Supplemental Table S3.** Quality of evidence (GRADE approach).

| Outcome | Number of studies | Participants of  High FiO_2_ group | Participants of  Low FiO_2_ group | Quality assessment | | | | | Quality of evidence |
| --- | --- | --- | --- | --- | --- | --- | --- | --- | --- |
|  |  |  |  | Risk of bias | Inconsistency | Indirectness | Imprecision | Publication bias |  |
| Surgical site  infection | 27 | 8010 | 7967 | Serious | Serious | Not serious | Not serious | Unlikely | ⊕⊕⊝⊝ Low |
| Short-term  mortality | 12 | 6071 | 6051 | Not serious | Serious | Not serious | Serious | Serious | ⊕⊝⊝⊝ Very low |
| Length of  hospital stay | 11 | 1866 | 1837 | Serious | Serious | Not serious | Serious | Unlikely | ⊕⊝⊝⊝ Very low |
| Myocardial injury | 8 | 2417 | 2428 | Not serious | Not serious | Not serious | Serious | Unlikely | ⊕⊕⊕⊝ Moderate |
| Atelectasis | 7 | 2611 | 2643 | Not serious | Very serious | Not serious | Serious | Unlikely | ⊕⊝⊝⊝ Very low |
| Organ-space SSI | 8 | 1047 | 1048 | Not serious | Not serious | Not serious | Serious | Unlikely | ⊕⊕⊕⊝ Moderate |
| Anastomotic leakage | 6 | 4146 | 3741 | Not serious | Very serious | Not serious | Not serious | Unlikely | ⊕⊕⊝⊝ Low |
| Pneumonia | 6 | 2410 | 2449 | Not serious | Serious | Not serious | Serious | Unlikely | ⊕⊕⊝⊝ Low |
| Reoperation | 6 | 1337 | 1353 | Not serious | Serious | Not serious | Serious | Unlikely | ⊕⊕⊝⊝ Low |

FiO_2_ = inspired oxygen concentration, SSI = surgical site infection.

Organ-space SSI is a class of surgical site infection where organs or spaces, other than the incision, opened or manipulated during surgery are involved.^4^

High quality means that we are very confident that the true effect lies close to that of the estimate of the effect.

Moderate quality means that we are moderately confident in the effect estimate: The true effect is likely to be close to the estimate of the effect, but there is a possibility that it is substantially different.

Low quality means that our confidence in the effect estimate is limited: The true effect may be substantially different from the estimate of the effect.

Very low quality means that we have very little confidence in the effect estimate: The true effect is likely to be substantially different from the estimate of effect.

Evaluation criteria are as follows:

Risk of bias was evaluated as “serious” if at least one trial assessed as having a high risk of bias was included.

Inconsistency was rated based on the heterogeneity statistic I^2^. It was judged “not serious” when I^2^ measured below 25%, “serious” when it was between 25-75%, and “very serious” when it exceeded 75%.

No trials failed to conform to our predefined inclusion criteria, nor did any details of intervention deviate from our premise. All outcomes were measured in straightforward manifestations. Therefore, indirectness was determined “not serious.”

For imprecision, both the confidence interval and sample size were put to consideration. If the confidence interval for risk ratio exceeded 2.00 and included both benefits and harms, the outcome was deemed to have a “very serious” risk of imprecision. If the confidence interval exceeded 1.00 or if it included both benefits and harms, imprecision was judged to be “serious.”

Publication bias was estimated using the Egger test for outcomes reported in 10 or more trials, namely SSI, short-term mortality, and length of hospital stay. A *p*-value of less than 0.05 was considered to indicate the possibility of a small-study effect.

**Supplemental Table S4.** Sensitivity analysis was performed (1) applying the random-effects model for all study outcomes, (2) according to the different definitions of surgical site infection, and (3) based on compliance with WHO guidelines.

| (1) Random-effects model |  |  |  |  |  |
| --- | --- | --- | --- | --- | --- |
| Outcomes | Events, High FiO_2_, n/N | Events, Low FiO_2_, n/N | Risk ratio (95% CI) | *P* value | I^2^ |
| Surgical site infection | 730 / 8010 | 839 / 7967 | 0.85 (0.73, 0.98) | 0.03 | 49 |
| Short-term mortality | 79 / 6190 | 76 / 6171 | 0.90 (0.57, 1.44) | 0.67 | 34 |
| Myocardial injury | 108 / 2417 | 121 / 2428 | 0.91 (0.72, 1.15) | 0.42 | 0 |
| Atelectasis | 326 / 2611 | 296 / 2643 | 1.12 (0.67, 1.86) | 0.68 | 88 |
| Organ-space SSI | 42 / 1047 | 50 / 1048 | 0.86 (0.58, 1.29) | 0.46 | 0 |
| Anastomotic leakage | 61 / 4146 | 88 / 3741 | 0.37 (0.15, 0.94) | 0.04 | 83 |
| Pneumonia | 106 / 2410 | 121 / 2449 | 0.88 (0.59, 1.31) | 0.52 | 46 |
| Reoperation | 128 / 1337 | 129 / 1353 | 0.96 (0.58, 1.59) | 0.88 | 32 |
| Outcome | High FiO_2_, Mean (SD) | Low FiO_2_, Mean (SD) | Mean Difference (95% CI) | *P* value | I^2^ |
| Length of hospital stay |  |  | 0.13 (-0.28, 0.54) | 0.53 | 66 |
| (2) Definition of surgical site infection | Events, High FiO_2_, n/N | Events, Low FiO_2_, n/N | Risk ratio (95% CI) | *P* value | I^2^ |
| ASEPSIS score definition^36,39,42,56,59^ | 21 / 490 | 49 / 486 | 0.43 (0.26, 0.70) | 0.0007 | 0 |
| CDC definition^31-33,37,38,40,43-45,47,49,53-55,57,58^ | 604 / 6814 | 697 / 6787 | 0.87 (0.79, 0.96) | 0.006 | 34 |
| (3) Compliance with WHO guidelines | Events, High FiO2, n/N | Events, Low FiO2, n/N | Risk ratio (95% CI) | P value |  |
| Yes^32,33,37,42-55,59^ | 225 / 4113 | 235 / 4061 | 0.84 (0.75, 0.94) | 0.002 | 54 |
| No^31,36,38-41,56-58^ | 709 / 8010 | 811 / 7967 | 0.94 (0.79, 1.12) | 0.52 | 35 |

FiO_2_ = inspired oxygen concentration, SSI = surgical site infection.

Organ-space SSI is a class of surgical site infection where organs or spaces, other than the incision, opened or manipulated during surgery are involved.^6^

**Supplemental Table S5.** Sensitivity analysis after removing all trials by Schietroma et al.^52-54^

| Outcomes | Events, High FiO_2_, n/N | Events, Low FiO_2_, n/N | Risk ratio (95% CI) | *P* value | I^2^ |
| --- | --- | --- | --- | --- | --- |
| Surgical site infection | 687 / 7763 | 753 / 7719 | 0.91 (0.83, 1.00) | 0.05 | 39 |
| Short-term mortality | 72 / 5943 | 60 / 5923 | 1.21 (0.86, 1.69) | 0.28 | 31 |
| Myocardial injury | 108 / 2256 | 118 / 2265 | 0.92 (0.73, 1.16) | 0.47 | 0 |
| Atelectasis | 326 / 2611 | 296 / 2643 | 1.11 (0.96, 1.28) | 0.16 | 88 |
| Anastomotic leakage | 48 / 3640 | 60 / 2613 | 0.80 (0.55, 1.16) | 0.24 | 0 |
| Reoperation | 121 / 1209 | 119 / 1225 | 1.04 (0.82, 1.31) | 0.75 | 0 |

FiO2 = inspired oxygen concentration, SSI = surgical site infection.

**Supplemental Figure S1.** Risk of bias assessment of the included studies (version 2.0).


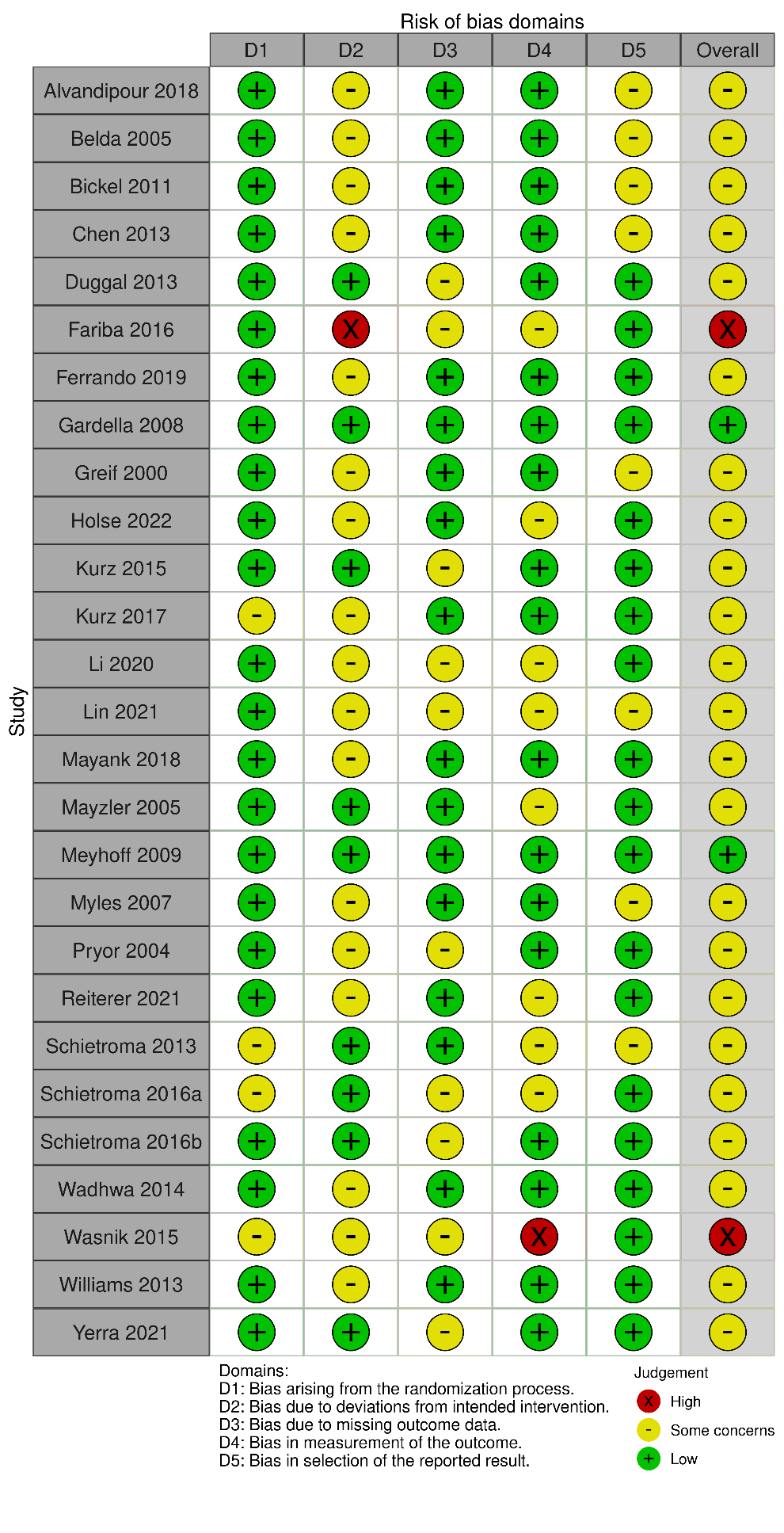


**Supplemental Figure S2.** Funnel plots of the following outcomes: A = surgical site infection, B = length of hospital stay, C = short-term mortality, D = myocardial injury, E = atelectasis. Each dot represents one study. The blue vertical dashed line is the pooled risk ratio (RR) or mean difference (MD). Diagonal dashed blue lines define a funnel within which 95% of the studies should lie in the absence of heterogeneity or selection bias.

**
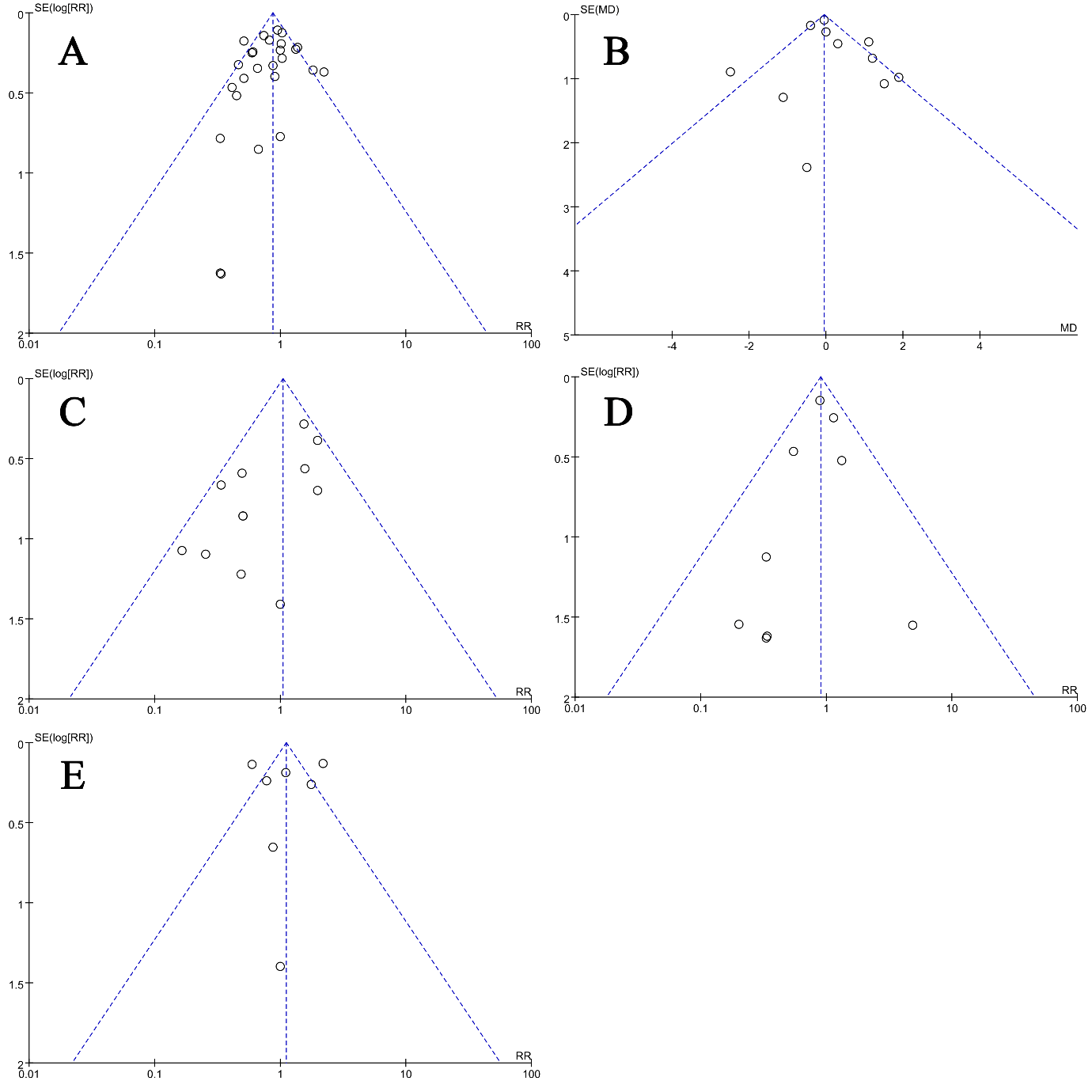
**

**Supplemental Figure S3.** Forest plot of comparison between high FiO_2_ vs. low FiO_2_: length of hospital stay. FiO_2_ = fraction of inspired oxygen.

**
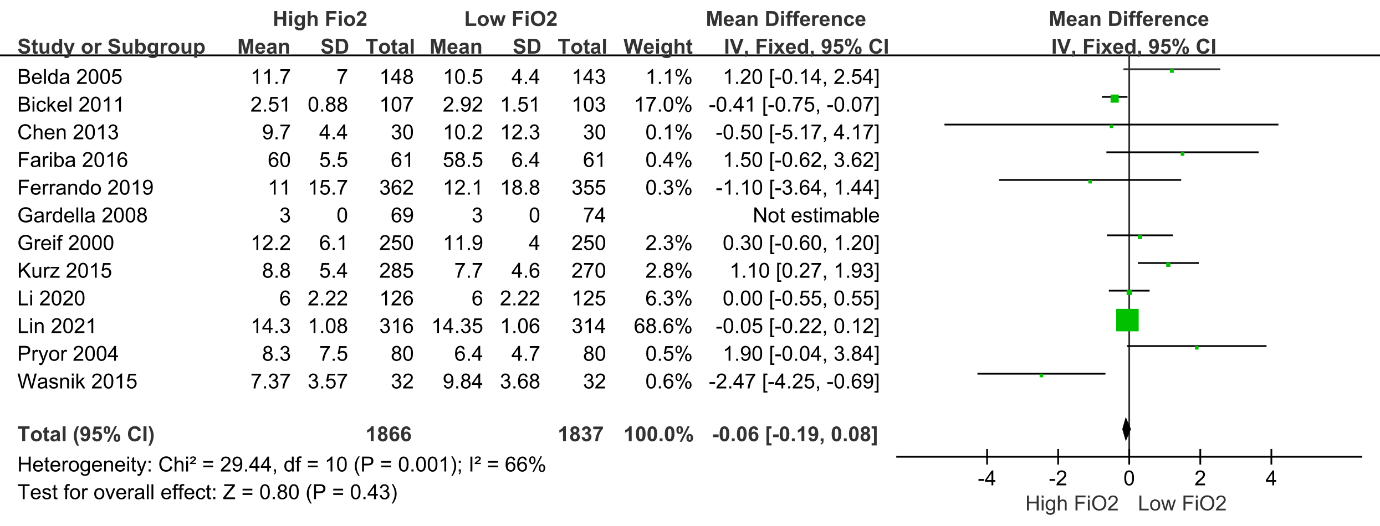
**

**Supplemental Figure S4.** Bubble plot for meta-regression of surgical site infection and covariates: A = age, B = percent of males, C = body-mass index, D = percent of patients with diabetes mellitus, E = duration of surgery, F = percent of current smokers. Each buble represents one study. The red lines mean the linear regression function for each covariate.


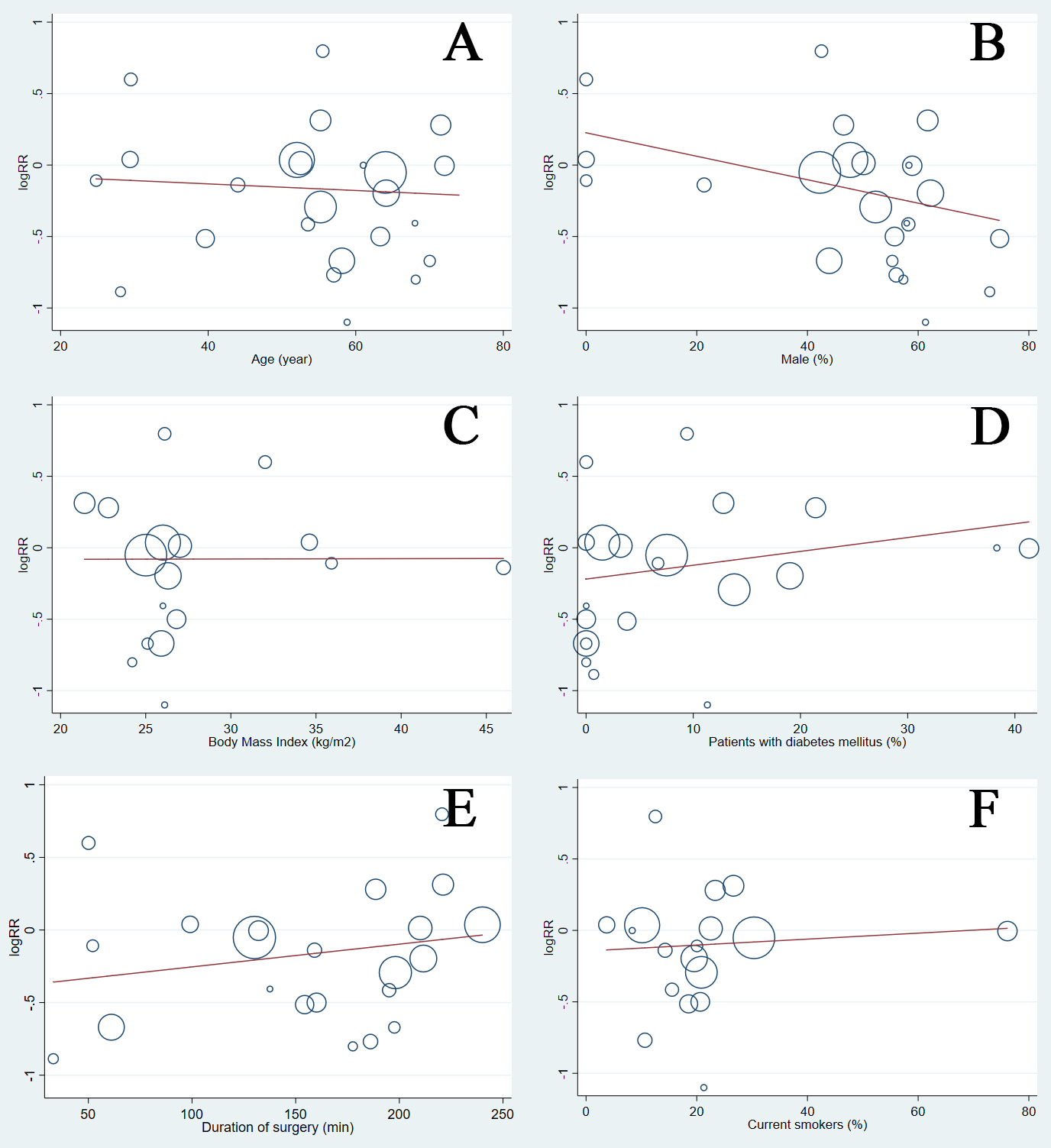


**Supplemental Figure S5.** Trial sequential analysis for length of hospital stay. The blue line means the cumulative z-score curve and the boundaries favoring high or low FiO2 or area of futility are shown in red lines. The blue line means the cumulative z-score curve. The boundaries favoring high or low FiO2 or area of futility are shown in red lines.


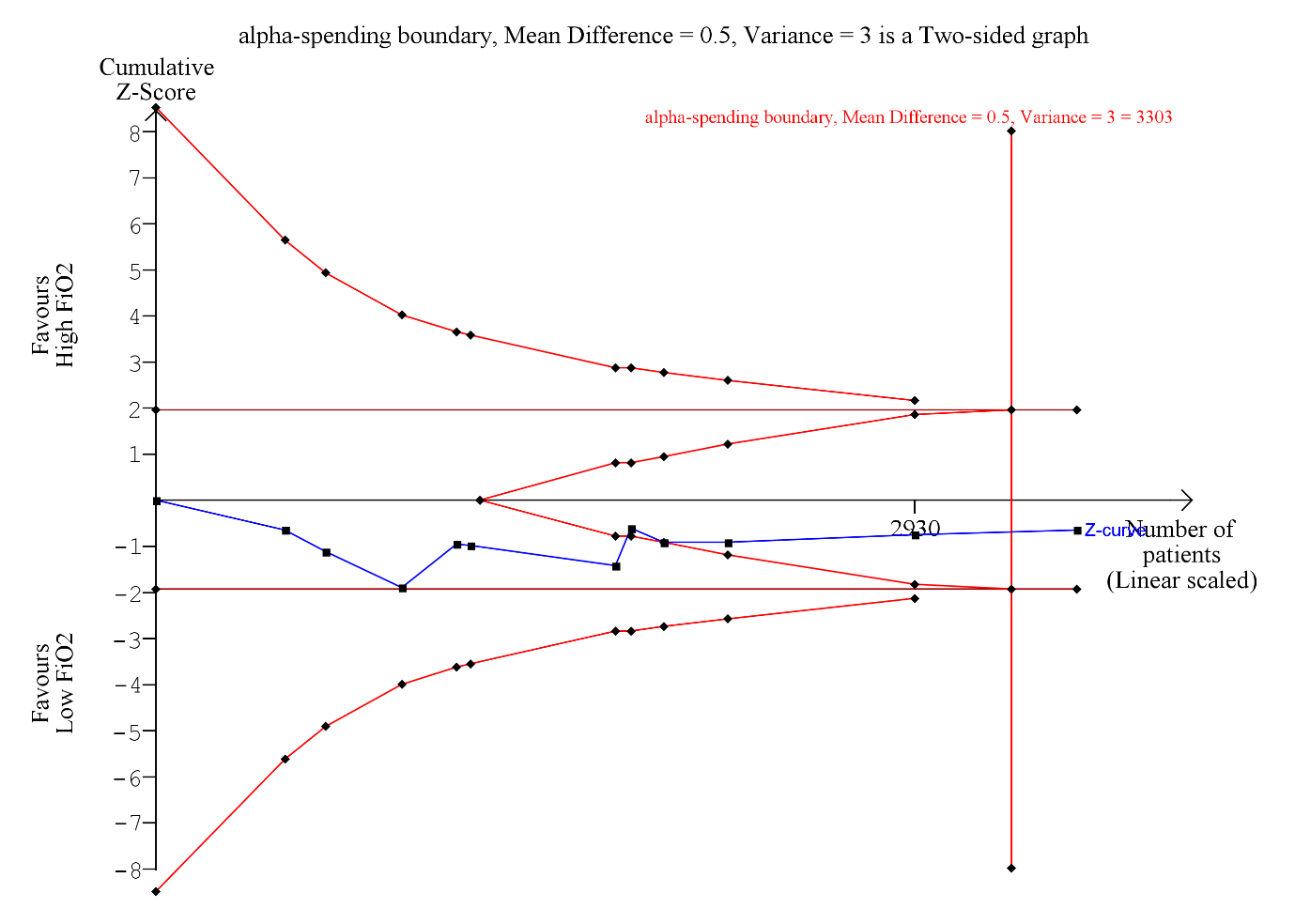


**Supplemental Figure S6.** Trial sequential analysis for short-term mortality. Pc = Probability in the control group, RRR = relative risk reduction, a = alpha error, b = beta-error. The blue line means the cumulative z-score curve. The boundaries favoring high or low FiO2 or area of futility are shown in red lines.


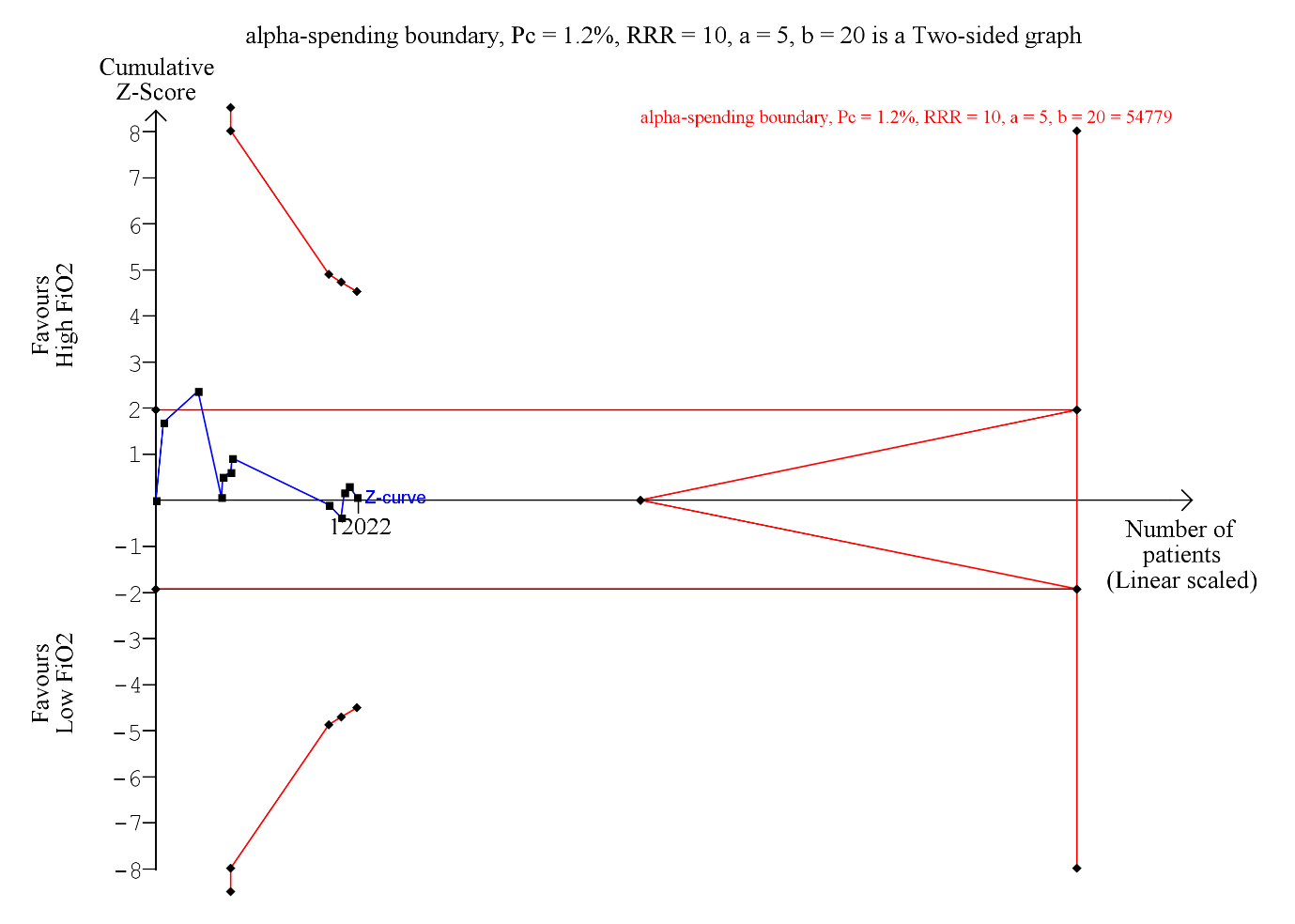


**Supplemental Figure S7.** Trial sequential analysis for myocardial injury. Pc = Probability in the control group, RRR = relative risk reduction, a = alpha error, b = beta-error. The blue line means the cumulative z-score curve. The boundaries favoring high or low FiO2 or area of futility are shown in red lines.


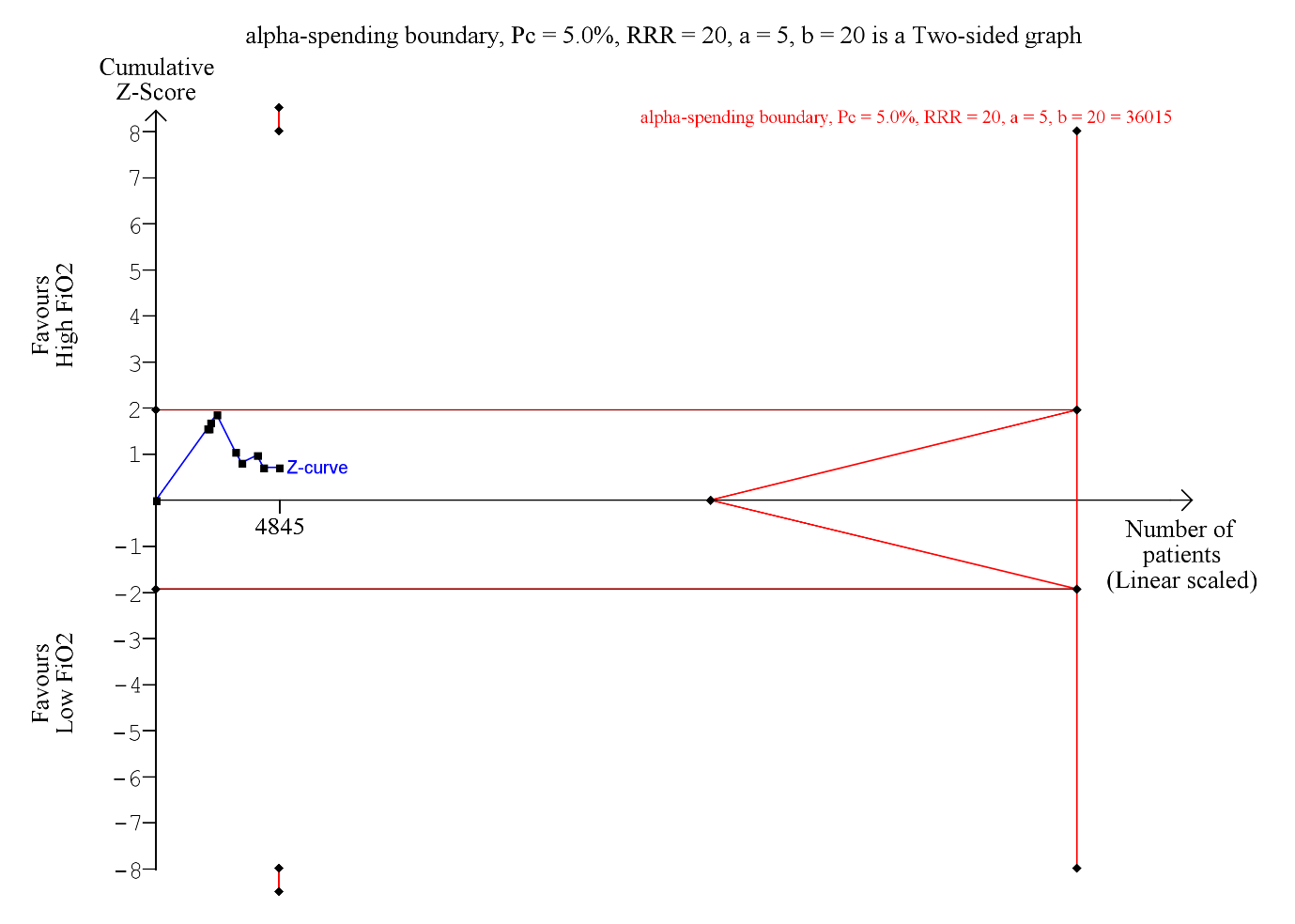


**Supplemental Figure S8.** Trial sequential analysis for atelectasis. Pc = Probability in the control group, RRR = relative risk reduction, a = alpha error, b = beta-error. The blue line means the cumulative z-score curve. The boundaries favoring high or low FiO2 or area of futility are shown in red lines.


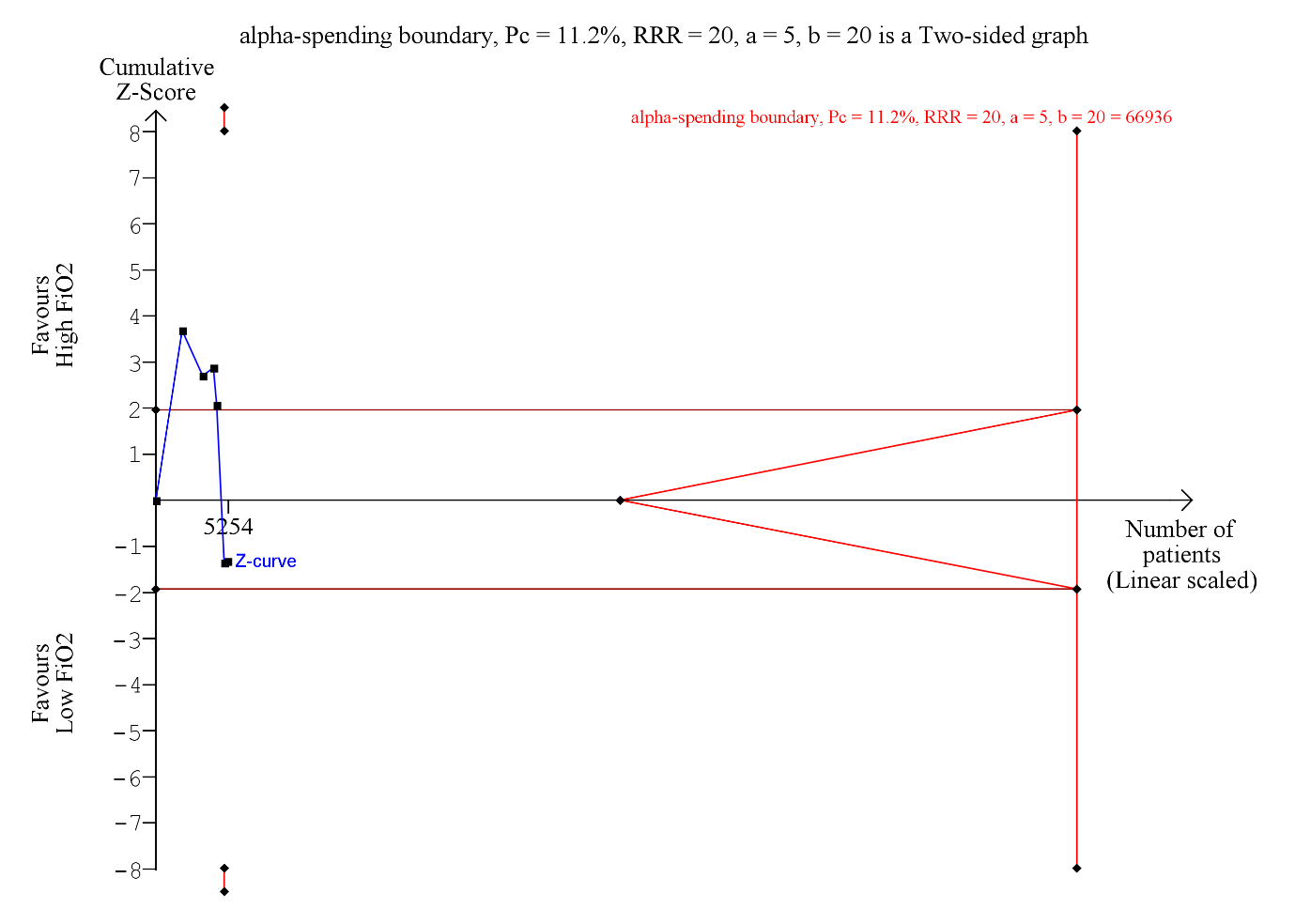

Supplement: Supplementary file 1 — Supplementary Information. [file 41598_2023_41300_MOESM1_ESM.docx]
